# Based on billions of words on the internet, people = men

**DOI:** 10.1126/sciadv.abm2463

**Published:** 2022-04-01

**Authors:** April H. Bailey, Adina Williams, Andrei Cimpian

**Affiliations:** 1 Department of Psychology, New York University, 6 Washington Place, New York, NY 10003, USA.; 2 Facebook Artificial Intelligence Research, Meta Platforms Inc., 770 Broadway, Floor 7, New York, NY 10003, USA.

## Abstract

Recent advances have made it possible to precisely measure the extent to which any two words are used in similar contexts. In turn, this measure of similarity in linguistic context also captures the extent to which the concepts being denoted are similar. When extracted from massive corpora of text written by millions of individuals, this measure of linguistic similarity can provide insight into the collective concepts of a linguistic community, concepts that both reflect and reinforce widespread ways of thinking. Using this approach, we investigated the collective concept person/people, which forms the basis for nearly all societal decision- and policy-making. In three studies and three preregistered replications with similarity metrics extracted from a corpus of over 630 billion English words, we found that the collective concept person/people is not gender-neutral but rather prioritizes men over women—a fundamental bias in our species’ collective view of itself.

## Teaser

Based on billions of words on the internet, the concept of a person is not gender-neutral but instead prioritizes men.

## INTRODUCTION

Recent advances in natural language processing have enabled cognitive scientists to use large corpora of naturally produced language to characterize the content of, and relations between, human concepts at a scale that is unprecedented in the history of the field. The assumption underlying this language-based approach to the study of concepts is surprisingly simple: Words that are used in similar contexts express concepts that are similar in content (*1*, *2*). The development of sophisticated tools for computing word-usage similarity from massive corpora of language (*3*–*7*) has thus opened the door for the study of what we call “collective concepts”—representations extracted from the aggregated linguistic output of millions of individuals that both reflect and reinforce widespread ways of thinking [(*8*–*10*); for a recent discussion, see (*11*)]. Here, we apply this approach to a corpus of over 630 billion words to characterize perhaps the most basic concept in human psychology, the concept of person (or people). How do collective concepts represent the human species? Are certain groups privileged over others in these representations? In three studies and three preregistered replications, we find a fundamental bias: The collective concept person is more similar to man than it is to woman. Given the fact that women and men each make up ~50% of our species (*12*), the finding that people are conflated with men at the level of collective concepts has many problematic consequences not only cognitively but also with respect to societal decision- and policy-making.

### Language and collective concepts

In this research, we used a natural language processing tool called “word embeddings.” Briefly, a word embedding is a high-dimensional vector that represents, in a compressed format, a word’s patterns of co-occurrences with the other words in a given corpus. Thus, the similarity between word embeddings, computed as the cosine of the angle between them in vector space, reveals the extent to which the corresponding words tend to be used in similar ways [i.e., in similar linguistic contexts; (*6*)]. For instance, the embeddings for words that are used almost interchangeably (e.g., “scientist” and “researcher”) are more similar than the embeddings for words that are only occasionally used in the same linguistic contexts (e.g., “scientist” and “smart”), which, in turn, are more similar than the embeddings for words that occur in very different contexts (e.g., “scientist” and “instead”). Precisely, “scientist” is more similar to “researcher” (0.767) than it is to “smart” (0.204) and to “instead” (0.036), where the highest possible similarity score is 1 [based on cosine similarity and fastText word embeddings; (*13*)]. By allowing us to measure similarity in word use, word embeddings provide a linguistic tool for approximating the similarity between the concepts being denoted.

The claim that similarity in word use can be used to measure similarity in concepts is motivated by the distributional hypothesis of word meaning, according to which words that occur in similar linguistic contexts have similar meanings [(*1*); see also (*2*, *14*)]. Linguist J. R. Firth summarized this hypothesis as, “You shall know a word by the company it keeps” [(*15*), p. 11]. To make the intuition behind this hypothesis concrete, consider a hypothetical situation in which a speaker uses the unfamiliar word “balak” (*16*). While a listener might not be familiar with this word, they can start to understand its meaning by paying attention to the linguistic context in which this word is used. For example, if the speaker says, “Each morning, Joe boiled water in the balak for tea,” the listener might start to guess that “balak” means something similar to “kettle” because the words alongside “balak”—“tea,” “boiled,” and “water”—also frequently co-occur with “kettle” in other contexts. Essentially, this is the principle that motivates the use of word embeddings. Word embeddings capture a word’s patterns of co-occurrences with other words to represent word meaning [broadly construed; see (*2*, *14*)]. In addition, because words denote concepts, word embedding vectors can be described equally validly as proxies for word meaning and as proxies for the concepts denoted by words.

When extracted from massive corpora of billions of words written by millions of individuals, word embeddings can be used to investigate collective concepts—concepts that both reflect and reinforce shared ways of thinking among a linguistic community. The notion of a collective concept, as we use it here, draws heavily on sociological theories about collective (*8*) or social representations (*9*). These are systems of concepts, values, and practices that characterize a community and that also go beyond (rather than being wholly reducible to) just what individuals in that community think. Our term “collective concept” thus refers to a collective or social representation that pertains to a concept (e.g., person).

This simple, language-based method of investigating collective concepts has already produced some remarkable results (*17*–*19*). For instance, using nothing more than similarity computations over word embeddings, researchers have been able to reconstruct the taxonomic structure of collective concepts [e.g., that wrist and ankle are the same kind of thing, and different kinds of things than dog or Hawaii; (*20*)] and the social biases embedded in them [e.g., that science is more similar to men than to women; (*11*, *21*–*23*)]. Here, we apply this powerful technique to a massive linguistic corpus to investigate the collective concept of person and its relation to its gender-specific counterparts, woman and man.

### The people = men hypothesis

Theories in philosophy, sociology, and linguistics have long argued that men are treated as the “default” humans, whereas women are treated as a gendered deviation from this male default [e.g., (*24*–*27*)]. Using the terminology of the present research, this argument can be translated into an empirical claim that the similarity between the collective concepts of people and men, which we will denote as Sim(people, men), is greater than the similarity between the collective concepts of people and women, which we will denote as Sim(people, women).

Empirical investigations in psychology have tended to support this people = men claim at the level of individuals’ concepts. For instance, lay participants describe more men than women when asked to think of examples of a person (*28*–*30*), select men more often than women to represent humanity as a whole (*31*), and are faster to associate men than women with words for people [(*32*); for a review, see (*33*)]. However, considering that the samples in these studies generally consisted of no more than a few hundred participants (and often fewer), the extent to which they provide insight into the collective concept of person is unclear.

Some larger-scale investigations, involving thousands to millions of participants, are relevant to our question. For instance, “he” occurs more often than “she” in the linguistic output of millions of individuals in news coverage and in published books (*34*, *35*). This overrepresentation of “he” is consistent with the people = men hypothesis. However, “he” may also appear more often than “she” because of the linguistic practice of referring to a person of unknown gender using “he” rather than “she”—that is, due to grammatical conventions rather than due to gender biases (*27*). Thus, previous large-scale investigations do not speak directly to biases in the collective concept person (and indeed they did not set out to do so) because they rely on simple frequency comparisons (e.g., does “he” occur more often than “she”?), whose interpretation is ambiguous. In contrast, word embeddings capture nuances in the typical linguistic contexts of words—including co-occurrences and higher-order co-occurrences (e.g., do “he” and “person” occur alongside the same words more often than “she” and “person”?)—and are thus ideally suited to investigate whether the collective concept of a person is more similar to man than it is to woman.

The present studies provide a direct investigation of the collective concept person—a concept that is not only central to the human experience but also the basis for nearly all health, safety, and workplace policy-making enacted in modern societies (*36*–*38*). Despite the importance of this concept, there has been far less research—and no large-scale research we know of—on gender bias in the concept of people. In contrast, other forms of gender bias (e.g., that science is more associated with men than with women) have been the focus of numerous large-scale studies involving thousands to millions of participants [e.g., (*39*)] as well as several meta-analyses [e.g., (*40*)]. The present studies fill this gap and investigate the collective concept people based on the aggregated linguistic output of millions of individuals. We hypothesize that the similarity between people and men will be greater than the similarity between people and women.

## RESULTS

To test whether Sim(people, men) > Sim(people, women) at the level of collective concepts, we used word embeddings (*13*) extracted from the May 2017 Common Crawl corpus [CC-MAIN-2017-22; (*41*)], which contains a large cross section of the internet: over 630 billion words from 2.96 billion web pages and 250 uncompressed TiB of content. Although the Common Crawl is not accompanied by documentation about its contents, it likely includes informal text (e.g., blogs and discussion forums) written by many individuals, as well as more formal text written by the media, corporations, and governments, mostly in English (*42*, *43*). Using word embeddings extracted from this massive corpus, we computed the similarity in linguistic context between words—a proxy for the similarity between the concepts denoted—as the cosine of the angle between corresponding embeddings in vector space, or cosine similarity.

### Study 1: Comparing words for people with words for women and men

In study 1, we conducted a straightforward test of the hypothesis that Sim(people, men) > Sim(people, women). We compared the similarity in linguistic context between words for people and words for men to the similarity in linguistic context between words for people and words for women. To do so, we first created suitable lists of words that denote the concepts people (e.g., “individual” and “humanity”; *n* = 30), women (e.g., “she” and “female”; *n* = 38), and men (e.g., “he” and “male”; *n* = 36; for examples, see Table 1; for full lists, see the Supplementary Materials). Second, we retrieved the word embeddings extracted by a standard algorithm [fastText with 300 dimensions; (*13*)] and computed the cosine similarities between the embeddings for (i) the words for people and the words for men and (ii) the words for people and the words for women.

**Table 1.** Summary of word lists across studies.

**Word type**	**Study**	**Gender stereotypicality**	**Examples**	***N***
Words for people	1		People, person, somebody, someone, human, humanity	30
Words that describe people (traits)	2A	Stereotypical of women	Accommodating, cheerful, fault-finding, gullible, opinionated, sympathetic	538
		Stereotypical of men	Abusive, candid, forward, grumpy, outspoken, unaffectionate	
	2B	Stereotypical of women	Appreciative, complicated, family-oriented, gentle, outgoing, suggestive	178
		Stereotypical of men	Arrogant, controlling, forceful, greedy, rational, witty	
Words that describe people (verbs)	3	Female-biased	Adore, complain, entertain, gossip, kiss, scare	252
		Male-biased	Appoint, cheat, honor, kill, respect, speak	
Words for women	1–3		Woman, women, female, females, she, ms	38
Words for men	1–3		Man*, men, male, males, he*, mr	36

*These so-called masculine generic terms are sometimes used generically to refer to a person of any gender. Key for our purposes, the present findings are not merely due to these words being in our word list: Similar results are obtained when these words are removed from the analyses (see the Supplementary Materials).

**Fig. 1.**
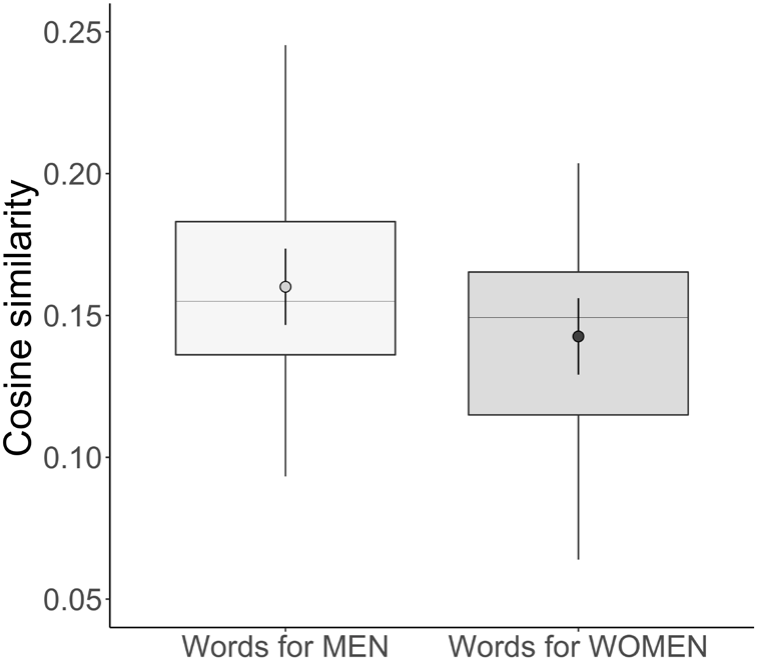
Cosine similarity between words for people, women, and men in study 1. Words for people were used in more similar contexts to words for men than to words for women, as indicated by the cosine similarities between the corresponding word embeddings. Embeddings for words that are always used in the same context approach a cosine similarity of 1, and embeddings for words that are never used in the same context approach a cosine similarity of 0. Boxplots show the full range of the raw data as well as the 25th and 75th percentiles (the bottom and top edges of the boxes, respectively), and the median is shown as a horizontal gray line. Dots are the fitted means, and error bars are 95% confidence intervals based on the fitted SEs.

We found that words for people were more similar in their use to words for men than to words for women, *B* = 0.017, SE = 0.004, *P* < 0.001, *d* = 0.465 (Fig. 1). Differences of this magnitude (*d* = 0.465) are considered “medium” by conventional standards for effect sizes [*d* = 0.50, (*44*); *d* = 0.36, (*45*)], and by comparison, some gender-stereotypical associations found in collective concepts are larger [e.g., science = men/arts = women, *d* = 1.24; (*21*)]. In summary, the collective concept people—measured with word embeddings extracted from a large cross section of the internet—overlaps more with the concept men than with the concept women.

### Study 2A: Comparing trait words descriptive of people with words for women and men

**Fig. 2.**
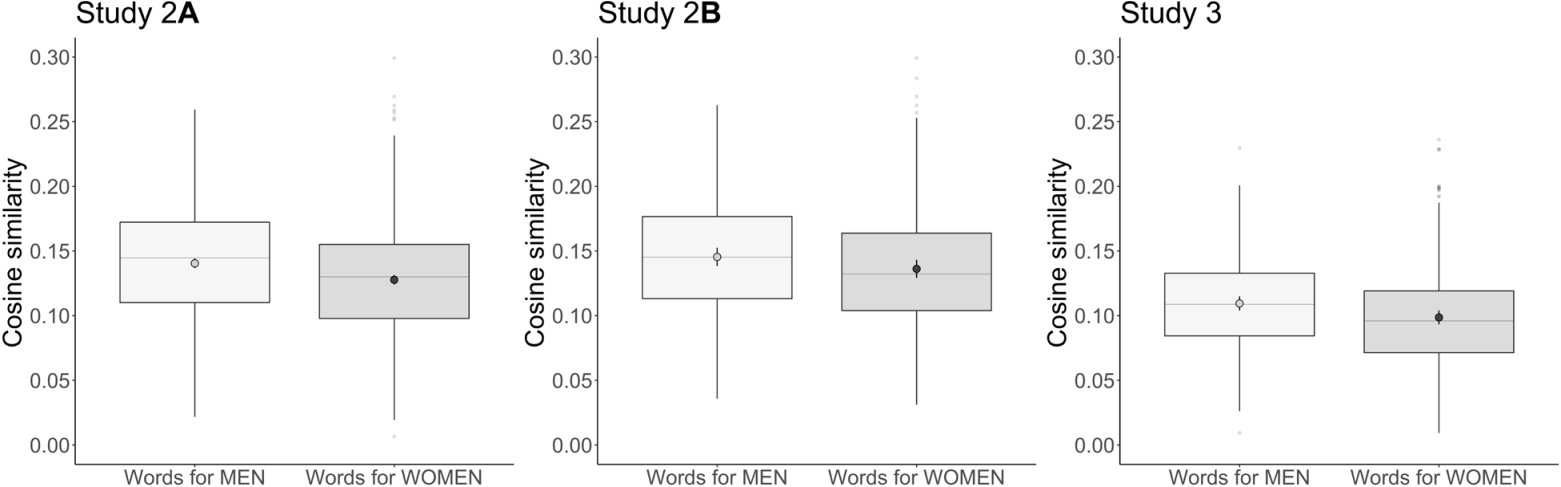
Cosine similarity between words for women and men and trait words in study 2A, trait words in study 2B, and verbs in study 3. Traits and verbs that describe what people are like and what they do were used in more similar linguistic contexts to words for men than to words for women. Embeddings for words that are always used in the same context approach a cosine similarity of 1, and embeddings for words that are never used in the same context approach a cosine similarity of 0. Boxplots show the full range of the raw data as well as the 25th and 75th percentiles (the bottom and top edges of the boxes, respectively), and the median is shown as a horizontal gray line. Dots are the fitted means, and error bars are 95% confidence intervals based on the fitted SEs.

Study 2 took a different approach to testing the hypothesis that Sim(people, men) > Sim(people, women). Instead of focusing on words for people, we investigated words denoting features central to this concept—specifically, words for traits that commonly describe what people are like. In study 2A, we compared 538 trait words identified in previous work as common descriptors of people [e.g., “extroverted”; (*46*)] to the same lists of words for women and words for men from study 1. We found that the linguistic contexts of these common person-descriptors were overall more similar to those of words for men than to those of words for women, *B* = 0.013, SE = 0.001, *P* < 0.001, *d* = 0.286 (Fig. 2, left). This difference is smaller than in study 1—likely because the trait words are more varied in meaning than the words for people—but is nevertheless statistically reliable and provides further evidence for the hypothesis that Sim(people, men) > Sim(people, women).

The hypothesis that Sim(people, men) > Sim(people, women) also licenses a striking prediction about gender-stereotypical associations. In previous work on individuals’ psychological stereotypes about women and men, gender stereotypes are often found to be symmetrical (*39*, *40*, *47*–*49*). For example, women are stereotyped to have communal traits such as compassionate more than agentic traits such as brave, whereas, conversely, men are stereotyped to have agentic traits more than communal traits (*40*). But in collective concepts, we predicted that gender-stereotypical associations would be asymmetrical. Our reasoning was as follows. If the collective concept of people is conflated with men (as in study 1), then words for men may appear in contexts that are similar to those of words for any trait that a person can display. Correspondingly, if the collective concept of women has less overlap with people (as in study 1), then words for women may appear in contexts that are similar to traits that are specifically stereotypical of women. That is, words denoting men may be similar in their usage to a wide range of common person-descriptor traits (e.g., both “brave” and “compassionate”), whereas words denoting women may be similar in their usage to a more specific set of person-descriptor traits that are stereotypical of women (e.g., “compassionate” rather than “brave”).

**Fig. 3.**
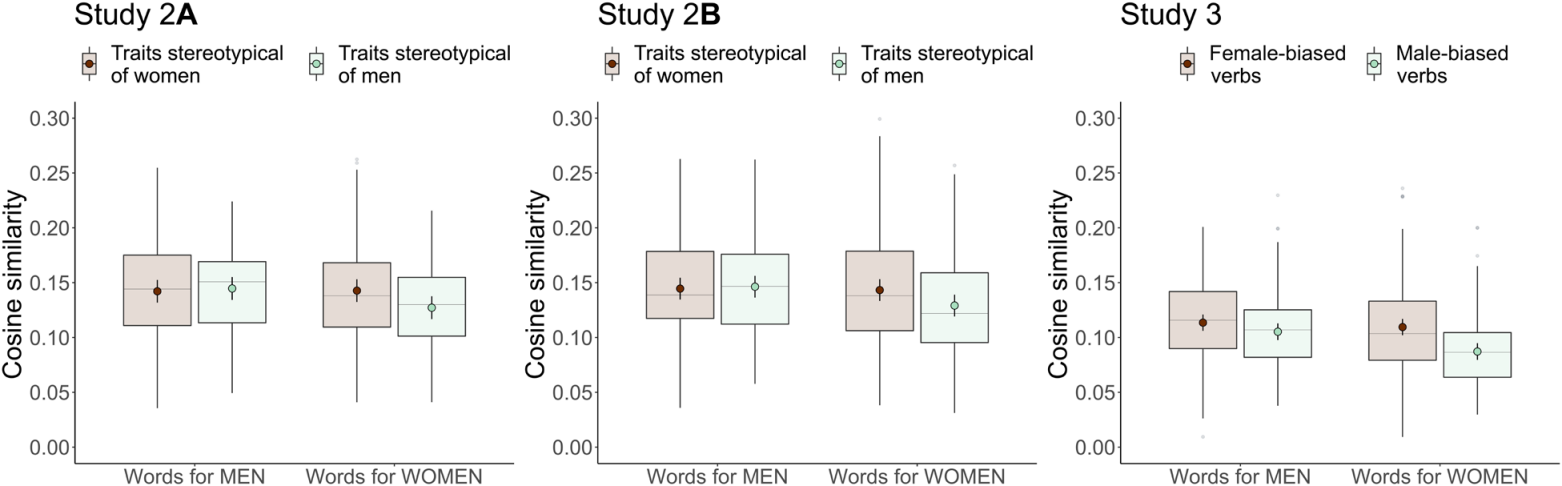
Cosine similarity between words for women and men and trait words in study 2A, trait words in study 2B, and verbs in study 3 as a function of gender stereotypicality. The cosine similarity between words for men and a wide range of traits and verbs did not differ based on previous gender stereotypicality designation, but words for women were used in more similar contexts to traits and verbs stereotypical of women than to traits and verbs stereotypical of men. Embeddings for words that are always used in the same context approach a cosine similarity of 1, and embeddings for words that are never used in the same context approach a cosine similarity of 0. Boxplots show the full range of the raw data as well as the 25th and 75th percentiles (the bottom and top edges of the boxes, respectively), and the median is shown as a horizontal gray line. Dots are the fitted means, and error bars are 95% confidence intervals based on the fitted SEs.

To test our prediction in study 2A, we first classified each trait word as stereotypical of women, men, or neither. Three raters who were unaware of our hypotheses rated the 538 traits; of these, 145 traits were rated by all three raters as more stereotypical of either women or men. Focusing on these 145 traits, we found an interaction between which gender was denoted (words for men versus words for women) and which gender the traits were rated as stereotypical of (stereotypical of men versus stereotypical of women), *B* = 0.018, SE = 0.004, *P* < 0.001. Specifically, the similarity in linguistic context between words for men and traits did not differ based on which gender the traits were rated as stereotypical of, *B* = 0.003, SE = 0.007, *P* = 0.733, *d* = 0.056. In contrast, words for women appeared in more similar linguistic contexts to trait words rated as stereotypical of women than to trait words rated as stereotypical of men, *B* = −0.016, SE = 0.007, *P* = 0.039, *d* = −0.344 (Fig. 3, left). Thus, we found an asymmetry in the gender-stereotypical associations embedded in collective concepts, as we predicted based on the hypothesis that Sim(people, men) > Sim(people, women).

### Study 2B: Conceptual replication of study 2A with a different set of trait words

The preceding study (study 2A) relied on person-descriptor traits rated for gender stereotypicality by just three raters. In study 2B, we extracted a list of 178 person-descriptor traits directly from the gender stereotyping literature in psychology (*40*, *47*–*50*). All 178 traits had been designated as stereotypical of either women or men based on ratings from thousands of participants. As in study 2A, these 178 person-descriptors were used in linguistic contexts that were overall more similar to those of words for men than to those of words for women, *B* = 0.009, SE = 0.002, *P* < 0.001, *d* = 0.194 (Fig. 2, middle).

In addition, we again found an interaction between which gender was denoted (words for men versus words for women) and which gender the traits were rated as stereotypical of (stereotypical of men versus stereotypical of women), *B* = 0.016, SE = 0.004, *P* < 0.001. That is, the gender-stereotypical associations reflected in collective concepts were again asymmetrical: The linguistic contexts of words for men did not differ in their similarity to the contexts of words for traits rated as stereotypical of women versus men, *B* = 0.002, SE = 0.007, *P* = 0.807, *d* = 0.036, but words for women were used in contexts that were more similar to words for traits rated as more stereotypical of women (versus men), *B* = −0.014, SE = 0.007, *P* = 0.049, *d* = −0.295 (Fig. 3, middle).

### Study 3: Comparing verbs descriptive of people with words for women and men

As a final test of the hypothesis that Sim(people, men) > Sim(people, women), study 3 followed the same logic as studies 2A and 2B but investigated verbs rather than trait words. If the collective concept people overlaps more with the concept men than with the concept women, then words that describe what people do and what is done to them (e.g., “love” and “annoy”) may also appear in more similar linguistic contexts to words denoting men than to words denoting women. We compared the cosine similarities between embeddings for 252 verbs that take words for people as syntactic arguments (*51*) and embeddings for words for men versus words for women. Overall, these “person verbs” were more similar in their usage to words for men than to words for women, *B* = 0.011, SE = 0.001, *P* < 0.001, *d* = 0.264 (Fig. 2, right). This result provides additional support for the hypothesis that Sim(people, men) > Sim(people, women).

The person verbs in this sample had been previously tagged as showing either a “female bias” or a “male bias” (to use the original authors’ terms) with respect to their syntactic arguments based on whether they tended to modify women (e.g., the verb “giggle”) or men (e.g., the verb “kill”) on Wikipedia (*51*). We used this syntactic tagging for an additional test of whether the gender-stereotypical associations reflected in collective concepts are asymmetrical, as was the case for trait words in studies 2A and 2B. We found an interaction between which gender was denoted (words for men versus words for women) and the gender bias of the verb (male-biased versus female-biased), *B* = 0.014, SE = 0.002, *P* < 0.001. The words for men did not differ in how similar their linguistic contexts were to the contexts of male- and female-biased person verbs, *B* = −0.008, SE = 0.005, *P* = 0.128, *d* = −0.202, but words for women were more similar in their linguistic contexts to female-biased verbs than to male-biased verbs, *B* = −0.022, SE = 0.005, *P* < 0.001, *d* = −0.544 (Fig. 3, right).

### Replication studies, control analyses, and robustness checks

Across studies 1 to 3, our findings were robust to a variety of checks (for details, see the Supplementary Materials). First, they were not specific to a particular set of word embeddings: We replicated our results in three preregistered replication studies using an entirely different set of word embeddings [GloVe with 300 dimensions, trained on the Common Crawl; (*7*)]. Second, our findings were not specific to a particular corpus: We replicated our results using word embeddings trained on a corpus of biomedical research text and clinical notes (*52*) instead of general-purpose text on the internet (i.e., the Common Crawl, which was the focus of the main studies). This biomedical corpus is of particular interest in part because biases in biomedical research have direct implications for gender (in)equity in health (*37*). Third, our findings were not explained by the fact that some of the words in our list of words for men are masculine generic words, meaning that English speakers sometimes use these words (e.g., “he”) to refer to a person of unknown gender (*27*). When these words were removed from the analyses, we observed the same pattern of results. Fourth, more generally, our findings were not contingent on any particular word: We found similar results when we iteratively recomputed all of our analyses, each time removing a single word from our word lists (i.e., “leave one out” analyses).

Fifth, we built confidence in our finding of an asymmetry in gender-stereotypical associations by replicating seemingly symmetrical patterns of association from previous work on collective concepts (*11*, *21*, *53*). Previous work has used a word-embedding association test (WEAT) to study gender-stereotypical associations in word embeddings (*21*). We applied this test to our data and replicated previous evidence for gender-stereotypical associations. However, because the WEAT was designed to mimic an influential test of human biases [the Implicit Association Test; (*54*)], it relies on a double difference score. That is, in the present case, the cosine similarity of each trait/verb and words for women is subtracted from the cosine similarity of that trait/verb and words for men and then this difference score for traits/verbs designated as stereotypical of women is subtracted from the difference score for traits/verbs designated as stereotypical of men (for formulas, see the Supplementary Materials). Double difference scores such as these preclude the possibility of observing the asymmetry in gender-stereotypical associations that we predicted and found.

In a sixth and final robustness check, we considered the possibility that disproportionately more text on the internet may be written about men than women, which could contribute to the people = men bias in collective concepts. The overrepresentation of men in text on the internet may itself be due to men being construed as the “default” person, but it could also be due to a variety of other factors [e.g., historic barriers to women’s participation in public roles; (*55*)]. Nevertheless, in the corpus from which the word embeddings we used were extracted, words for men did not occur significantly more often than words for women (for details, see the Supplementary Materials). Thus, frequency differences cannot explain the present finding that the collective concept of people is more similar to men than women. Even if words for men were, in fact, more frequent than words for women in our corpus, that would not necessarily explain our findings. Word embeddings tend to be more accurate for words that are more frequent (*56*), but a difference in precision between the embeddings for words for women and men would not, by itself, explain why the words for men were systematically more similar in usage to words for people. Put differently, the extra “noise” in the embeddings for words for women would have to be directional to explain our results. But to reiterate, we did not find evidence that words for men occurred at higher frequencies than words for women in the present corpus.

## DISCUSSION

We investigated the collective concept of person/people using computational tools applied to language from a large cross section of the internet (630+ billion words) and found that this concept is not gender-neutral but instead prioritizes men over women. A key contribution of these large-scale studies is to demonstrate that the people = men bias is embedded in our species’ collective view of itself and is thus likely to be pervasive. Based on the hypothesis that Sim(people, men) > Sim(people, women), we also predicted and found that the gender-stereotypical associations in collective concepts are asymmetrical. Whereas words for women were semantically closer to words for traits and actions stereotypical of women (versus men), words for men did not show the corresponding difference. That is, the collective concept of women is specifically associated with the traits and actions stereotypical of women, but men is associated with a broader range of person-descriptive traits and actions.

The present results contribute to the extensive literature on stereotypes in psychology. Gender stereotypes are often found to be symmetrical: Men are thought to be agentic (e.g., brave) more than communal, and women are thought to be communal (e.g., compassionate) more than agentic [e.g., (*40*)]. But we find that gender-stereotypical associations reflected in collective concepts are asymmetrical. What explains this difference?

One possibility is suggested by the fact that stereotypes and collective concepts are distinct types of representations. According to a definition common among psychologists, stereotypes are individuals’ beliefs that a certain social group has or lacks a certain attribute [e.g., (*40*)]. In contrast, while a collective concept reflects, to some extent, the beliefs of individuals in the relevant community, it is also by definition not just the sum of these beliefs (*8*, *9*). Collective concepts, as measured through word embeddings, likely capture not just individuals’ beliefs but also ideas that transcend individuals and are enmeshed in broader social systems and historical traditions. In summary, one reason why collective concepts and stereotypes show different patterns of gender-stereotypical associations (respectively, asymmetrical and symmetrical patterns) may be because they are two distinct types of representations.

In addition, the ways in which collective concepts and stereotypes are measured may help explain their different patterns of gender-stereotypical associations. Conventional ways of measuring gender stereotypes make gender salient to participants by asking questions that directly contrast women and men: for example, “In general, do you think each of the following characteristics is more true of women or men, or equally true of both?” (*40*). In turn, the salience of gender may prompt participants to assign traits to women and men in a mutually exclusive fashion, resulting in more symmetrical patterns of gender stereotypes than might otherwise be observed. Even indirect measures of stereotypes [e.g., the Implicit Association Test; (*39*, *54*)] make gender salient to participants by having them sort women and men by gender group—these measures also tend to rely on double difference scores that hide any asymmetry, if present. In contrast, here, collective concepts were extracted from language produced in a broad range of real-world contexts, and in all likelihood, many of these naturalistic contexts did not make gender salient. Under these conditions, we found an asymmetrical pattern with greater gender-stereotypical associations concerning words for women than words for men. It will be important for future research to consider, and test, whether this asymmetry in gender-stereotypical associations in collective concepts may, in fact, also characterize individual-level gender stereotypes if they are measured without making gender salient to participants.

The present work suggests several additional avenues for future research as well. Here, we showed that women are less central than men to the collective concept people, but gender nonbinary individuals may be even more marginalized in this collective concept, given that the very existence and legitimacy of these identities have been questioned [(*57*, *58*); but see (*59*)]. Furthermore, words for women and men (e.g., “female” and “male”) apply to individuals with a range of other social identities besides gender, such as race, ethnicity, age, and nationality (*60*, *61*). Future research should consider possible intersections between gender (including nonbinary identities) and other key dimensions of identity in collective concepts. This could be done by examining embeddings for words that simultaneously encode information about gender and, for instance, race (e.g., first names). Such research could reveal whether the people = men bias is more pronounced about certain subgroups of people than about others.

In addition to examining variation in the people = men bias about various subgroups, it would also be worthwhile to examine variation of this bias among different groups and subgroups of speakers (e.g., men versus women, English speakers versus Spanish speakers, adults versus children, and people from the United Kingdom versus people from the United States). This could be done by examining word embeddings trained on a smaller corpus of language produced exclusively by members of a certain subcommunity. Such investigations of different subcommunities could also help address two open questions about the present phenomenon, which we discuss next.

First, is it possible that the people = men bias is driven largely by men? Men may write disproportionately more text on the internet compared to people with other gender identities, and men are also particularly likely to prioritize their own gender group in their individually held person concept (*32*). As a result, men’s linguistic output may be largely responsible for an overall people = men bias in the collective concept of a person. One of our robustness checks makes this possibility somewhat unlikely. Recall that we found virtually the same amount of people = men bias in word embeddings trained on a corpus of biomedical text. Given the overrepresentation of men as authors in the biomedical domain (*62*), this corpus presumably includes an even greater proportion of text written by men compared to undifferentiated text on the internet (i.e., the Common Crawl corpus). The fact that this (presumably) greater imbalance in the gender of the individuals who produced the text did not result in any appreciable change in the extent of people = men bias goes against the possibility that men alone are driving the patterns we observed here. Nevertheless, future research on smaller, more differentiated corpora (i.e., produced by women versus men) would be informative about the role of speakers’ own gender identity in the people = men bias.

A second open question is the following: Is it possible that the people = men bias documented here is driven by particular features of the English language? Languages differ in the extent to which their grammars encode information about gender. Some languages specify gender information on nouns, pronouns, verbs, and adjectives (e.g., Spanish); other languages do not include any information about gender in that way (e.g., Turkish); English falls somewhere in between. This variation across languages is potentially relevant to the people = men bias: The more a language encodes information about gender, the less likely it is to include suitable gender-neutral terms, and the more it may then license using male terms when referring to a person of unknown gender [e.g., “he” in English and “él” in Spanish; (*27*)]. The practice of using such masculine generic terms may be part of what causes the people = men bias to develop in collective concepts. It is noteworthy that the presence of masculine generic terms in our word lists did not explain the people = men bias in our own data; this bias was observed even when masculine generic terms were excluded from the analysis (see the Supplementary Materials). Nevertheless, it is possible that the very existence of masculine generics in a language exacerbates the people = men bias in collective concepts because masculine generics suggest to speakers of that language that one gender (i.e., men) can stand in for the generic person category. Variation in this aspect of language could thus correspond to variation in the people = men bias across different linguistic communities. Future research could systematically compare different linguistic communities while also accounting for other cultural-level variation in gender attitudes and norms to test this possibility. Such research would also contribute to a more complete view of who is privileged in the collective concept people among different linguistic communities around the world.

Collective concepts do not only reflect but also instill and reinforce widespread ways of thinking about women and men (*8*, *9*). Thus, the present findings have broad implications for society.

First, the conflation of people with men at the level of collective concepts likely helps to instill a people = men cognitive bias in each new generation of individuals. In the present investigation of collective concepts, we found the people = men bias in large-scale statistical regularities in the linguistic environment. Children are sensitive to the statistical structure of their linguistic environments (*16*, *63*, *64*). It is thus likely that children are able to infer how others in their linguistic community conceive of people without receiving any explicit input on this topic. In this way, the people = men bias is maintained across generations, perpetuating decision-making that advantages men with negative consequences for women’s health, safety, and workplace well-being (*36*–*38*).

Second, the people = men bias in word embeddings likely spills over into the wide range of downstream artificial intelligence applications that use word embeddings, including machine translation, automatic answering of user-generated questions, automatic recommendations on a range of topics (e.g., in the financial or legal system), and content ranking systems [e.g., Google Search and Twitter feed ranking; (*65*, *66*)]. Previous research has documented social biases in virtually all applications that are reliant on word embeddings [e.g., (*67*–*70*)]. Consider machine translation, for example. When “the doctor” in the English sentence “The doctor asked the nurse to help her in the procedure” is translated into Spanish, this noun is automatically assigned masculine gender, although the pronoun “her” in the original sentence clearly indicates that the doctor was a woman [“El doctor le pidio a la enfermera que le ayudara con el procedimiento”; (*71*)]. Such gender biases in machine translation have been documented in currently active commercial systems that rely on word embeddings (*72*). Ongoing efforts to “debias” word embeddings to prevent them from replicating such biases have yielded mixed results (*56*, *73*, *74*) and have yet to consider the fundamental people = men bias we uncover here. This raises a key point. Even if every single individual’s own cognitive bias to conflate people with men were to suddenly disappear, there would still be people = men bias in our culture because it is embedded in our artificial intelligence systems and applications that are built on the linguistic output of previous generations. We hope that the present work guides future efforts to debias natural language processing algorithms.

To conclude, we investigated the collective concept of people using word embeddings distilled from billions of words on the internet. We found that speakers write (and to some extent presumably, think) about people and men more similarly relative to how they write (and think) about people and women, indicating that the collective concept people privileges men over women.

## MATERIALS AND METHODS

In all studies, our methods proceeded in three steps. In step 1, we created suitable lists of words for the concepts of interest. In step 2, we extracted word embeddings for each word on these lists. In step 3, we computed cosine similarity scores—a standard metric of similarity in word embeddings. Steps 2 and 3 are the same across studies and are thus only described in detail under study 1. Note that throughout, we use small caps to distinguish concepts from words, following a long-standing convention in cognitive psychology (e.g., people is the concept denoted by the word “people”). We also assume that singular and plural versions of the same word (e.g., “person” and “people”) denote the same substantive concept. We thus use the singular and plural words interchangeably when referring to concepts (e.g., person and people).

### Study 1

#### Word lists (step 1)

We first generated lists of words for the concepts people, women, and men. For people, a preliminary list was developed by the research team. For women and men, we used the gender dictionaries (i.e., word lists) supplied by the Linguistic Inquiry and Word Count software [LIWC2015; (*75*)] as a starting point. We removed gender words that pertained to specific domains with gender-stereotypical connotations (e.g., personal relationships and leadership), focusing as much as possible on words for men and words for women as generic constructs. Note that the present investigation focuses only on the gender concepts of women and men. Our methodology does not isolate representations of gender nonbinary individuals (*76*), nor does it differentiate between biological and social aspects of sex and gender [see gender/sex; (*77*)]. Our three lists of words for the concepts people, women, and men were further augmented with synonyms and highly related words by inputting each word into WordNet (*78*). This process resulted in preliminary lists of 28 words for people, 33 words for women, and 32 words for men.

Six coders who were unaware of our hypotheses rated these preliminary lists. Each list was presented in a separate block, with the order of the blocks randomized, although the gender blocks were always completed back-to-back. For each of the three types of words, coders were provided with a description of the underlying concept and then rated each word in terms of its fit with this concept (1 = not a good fit to 9 = a good fit). The order of the words on each list was randomized. Intraclass correlations (ICCs) treating both raters and words as random effects indicated moderate consistency among coders, ICC = 0.65 (*79*). Ratings were generally high—no words were rated below the scale midpoint—and thus all words were retained. Coders were also asked to generate additional words that were a good fit for the concept but were not already included in the lists they rated. We added the three words that were generated by two or more coders (i.e., “beings” and “group” for people and “femme” for women).

Last, we again examined the resulting lists of words. At this stage, we added seven gender words that had an obvious other-gender counterpart but that the previous steps had not produced. For instance, the gender word list included “male’s” but not “female’s,” so we added “female’s” at this stage along with “guys,” “gentleman’s,” “manhood,” and “laddie” to words for men (to parallel “lady’s,” “womanhood,” and “lassie”) and “schoolgirls,” “womens,” and “shes” to the words for women (to parallel “schoolboys,” “mens,” and “hes”). This resulted in our final list of 30 words for people, 38 words for women, and 36 words for men. Several examples of each type of word are provided in Table 1; the full lists are available in the Supplementary Materials.

#### Word embeddings (step 2)

We used fastText—an unsupervised predictive learning algorithm—word embeddings that had been trained on the May 2017 Common Crawl corpus (*13*). Although fastText word embeddings are available for other, smaller corpora, we chose the Common Crawl because the present study investigated the people = men hypothesis in culture broadly rather than in a specific domain, so the largest available corpus was the best fit for our research aims. We extracted fastText embeddings with 300 dimensions for each word on our three lists.

The May 2017 Common Crawl is a large collection of over 630 billion tokens (roughly, words) and contains 2.96+ billion web pages and over 250 uncompressed TiB of content (*41*). Recent investigations of the Common Crawl suggest that most of this corpus is written in English and based on webpages generated within a year or two of their inclusion in the corpus (*43*). The most prevalent 25 websites in the 2019 version include websites on patent filings, news coverage, and peer-reviewed scientific publications (*43*), but more informal content such as travel blogs and personal websites are also represented (*42*).

#### Cosine similarity (step 3)

To measure similarity between word embeddings, we computed the cosine similarity between each word for people and each gender word [as in (*21*)]. Cosine similarity is the cosine of the angle between two vectors—in this case, two word embeddings. Similarity scores range from −1 to 1 and can be thought of as being conceptually similar to a correlation coefficient. A cosine similarity score of 1 indicates that the two words are used in identical contexts; a similarity score of 0 indicates that the two words are orthogonal and used in unrelated contexts; and a score of −1 indicates that the two words are used in exactly opposite contexts.

Following the analytic strategy of (*21*, *22*), we computed two averages for each word for people: (i) the average across the word’s cosine similarity scores with all words for women and, separately, (ii) the average across the word’s cosine similarity scores with all words for men. This process resulted in two scores for any given word for people (e.g., “person”): One score captured the average similarity between this word and words for women, and the other score captured the average similarity between this word and words for men. These scores allowed us to test the hypothesis that Sim(people, men) > Sim(people, women).

### Study 2A

The methods and materials were similar to study 1 and again proceeded in three steps. In step 1, we created a suitable list of person-descriptor trait words (*46*). The list of words for men and words for women was the same from study 1. In step 2, we extracted word embeddings for each word on these lists, using fastText word embeddings with 300 dimensions trained on the Common Crawl corpus. In step 3, we computed the average cosine similarity between each trait word and words for women and, separately, words for men.

To create a suitable list of common trait words that describe what people are like, we drew on the literature in personality psychology. An influential paper (*80*) developed several lists of traits that capture a range of basic aspects of people’s personalities. These lists have subsequently been used widely to study personality, including a list of 587 traits that was recently used by (*46*). Following precedent (*46*), we removed 47 amplifications (e.g., “overambitious”) from this list. We also removed the trait words “masculine” and “feminine” because these words were also in our list of words for women and words for men. For the present study, this process resulted in a final list of 538 traits.

Next, we determined which gender (if any) each trait was stereotypical of. By necessity, we made this determination using conventional methods that make gender salient to coders (see Discussion). Six coders who were unaware of our hypotheses rated the 538 traits as stereotypical of either women or men. Coders also had the option to say that a given trait was not specifically stereotypical of either women or men or that the word was unfamiliar to them. Because of the large number of traits, each coder only coded half of the traits, meaning that each trait was coded by three of the six coders. To be conservative, we designated traits as stereotypical of women or men only if there was consensus among the three coders. This occurred for 145 traits. Several examples of each type of trait are provided in Table 1; the full lists are available in the Supplementary Materials.

### Study 2B

The methods and materials were the same as in study 2A, except that we used a different list of person-descriptive trait words. To create this list, we drew on the gender stereotyping literature in psychology. Several investigations of gender-stereotypical beliefs both about the self and about others have identified lists of common descriptors—often traits—that are considered particularly characteristic of women or men. These designations are based on large-scale polling data as well as laboratory-based studies with U.S. and international participants.

We examined five such lists to extract an initial list of 316 words (*40*, *47*–*50*). Many traits appeared on multiple lists—as would be expected given how these lists are created—so we removed repetitions. Because our focus was on traits and trait-like descriptors, we also removed occupation nouns. For the purpose of extracting word embeddings, we removed multiword phrases or, whenever possible, split them into single-word descriptors; for instance, we changed “polite and well-mannered” into “polite” and “well-mannered” (*40*). Last, we removed the traits “masculine” and “feminine” because these words were in our list of words for women and words for men. This process resulted in a final list of 178 traits. The list of words for women and words for men was the same from study 1. Several examples of each type of trait are provided in Table 1; the full lists are available in the Supplementary Materials.

### Study 3

The methods and materials were the same as in studies 1 and 2, except that we compared the cosine similarity of words for women and, separately, words for men with a list of person-descriptive verbs. To create a suitable list of verbs, we drew on the natural language processing literature on gender bias. Specifically, a previous investigation (*51*) automatically extracted verbs based on whether they were more likely to take words for women (e.g., the verb “giggle”) or words for men (e.g., the verb “kill”) as syntactic arguments on Wikipedia. This process identified 300 instances of verbs that are relatively more “female-biased” or “male-biased,” to use the original authors’ terminology. These verbs are suitable for our purposes because they describe things that people (women and men) do and can thus be used as proxies for the concept people. Furthermore, the fact that these verbs were already designated as male- or female-biased enabled us to test our prediction of an asymmetry in gender-stereotypical associations reflected in collective concepts.

Note that some verbs appeared more than once on the original authors’ (*51*) list because their gender-bias designation depended on two other factors: the verb’s valence (i.e., sentiment) and the syntactic position of the gender-biased arguments (subjects versus objects). Verbs were designated as positive, negative, or neutral in valence, and some verbs had, for instance, positive connotations with arguments of one gender but neutral connotations with arguments of another gender. Verbs also could exhibit bias toward one gender in the subject position but toward another gender in the object position. For instance, the verb “create” was female-biased in the object position with positive connotations but male-biased in the subject position with neutral connotations.

Of the 300 verbs on the initial list, we removed verbs that were both male- and female-biased, as long as they also had the same valence in both cases and the bias occurred in the same syntactic position. We removed these verbs because our research question requires a list of verbs with distinct gender-stereotypical designations. For verbs that repeated in all respects except that they were found to have multiple valences (e.g., positive and neutral), we removed the nonneutral valence cases to avoid redundancies. Last, we removed a few items from the initial list that were not verbs or were otherwise ambiguous (e.g., “brazen” was removed because it is an adjective rather than a verb). This process resulted in a final list of 252 cases of verbs, corresponding to 211 unique verbs. As explained above, this list contained some repetitions based on differing valence or syntactic position of the gender bias (subject versus object). The list of words for men and words for women was the same from study 1. Several examples of verbs are provided in Table 1; the full lists are available in the Supplementary Materials.

## Supplementary Materials

### This PDF file includes:

Supplementary Text Figs. S1 to S4 Tables S1 to S8 References
