# Supplementary material for: Based on billions of words on the internet, people = men
Source: Sci Adv. 2022 Apr 1;8(13):eabm2463. doi: 10.1126/sciadv.abm2463 (PMC10938580; doi:10.1126/sciadv.abm2463)
Supplement: Supplementary file 1 — Supplementary Text Figs. S1 to S4 Tables S1 to S8 References [file sciadv.abm2463_sm.pdf]

Science Advances
AAAS

Supplementary Materials for

Based on billions of words on the internet, PEOPLE = MEN

April H. Bailey*, Adina Williams, Andrei Cimpian

*Corresponding author. Email: ab9490@nyu.edu

Published 1 April 2022, *Sci. Adv.* **8**, eabm2463 (2022)
DOI: 10.1126/sciadv.abm2463

This PDF file includes:

Supplementary Text
Figs. S1 to S4
Tables S1 to S8
References

Supplementary Materials for
Based on billions of words on the internet, PEOPLE = MEN

Study 1

Additional Methodological Details of the Findings Reported in the Main Text in Study 1

Our final word lists consisted of 30 words for PEOPLE (Table S1), 38 words for WOMEN (Table S2), and 36 words for MEN (Table S2).

Additional Analytic Details of the Findings Reported in the Main Text in Study 1

As reported in the main text, we found that generic words for PEOPLE were more similar in their usage to words for MEN (M = 0.16, SD = 0.04) than to words for WOMEN (M = 0.14, SD = 0.04), B = 0.02, SE < 0.01, p < .001, d = 0.47. This result was the output of a mixed-effects linear regression with gender (words for MEN vs. words for WOMEN; categorical variable) predicting cosine similarity to words for PEOPLE, with a random intercept for each word for PEOPLE.

All mixed-effects linear regressions were conducted using R and lmerTest; p values were obtained with the Satterthwaite's degrees of freedom method (*81*). Here and throughout, the d values are coefficients from regressions with standardized outcome variables. That is, the d values represent the mean differences between words for MEN and words for WOMEN in standard deviation units.

Table S1

List of Words for PEOPLE in Study 1 With Average Fit Ratings

	Coder rating		Coder rating		Coder rating
beings	-	individual	9.00	somebody	9.00
citizenry	5.17	individuals	9.00	someone	9.00
folk	7.00	masses	8.17	soul	8.17
folks	7.67	mortal	6.50	souls	7.17
group	-	mortals	6.83	their	8.83
human	9.00	multitude	5.67	them	8.83
humanity	9.00	multitudes	6.17	they	8.83
humankind	8.50	people	9.00	tribe	5.50
humanness	6.83	person	9.00	tribes	5.50
humans	9.00	somebodies	7.17	yall	8.00

Note. The words that do not have ratings were added after the rating study was conducted because of suggestions from the coders as described in the Materials and Methods sections of the main text.

Table S2

List of Words for WOMEN and Words for MEN in Studies 1-3 With Average Fit Ratings

Words for WOMEN				Words for MEN			
	Coder rating		Coder rating		Coder rating		Coder rating
female	8.33	lady's	8.67	boy	8.67	lad	6.33
female's	-	lass	6.17	boy's	8.33	laddie	-
females	8.33	lassie	6.00	boyhood	7.83	male	8.83
feminine	8.67	ma'am	8.33	boyish	7.67	male's	8.33
femininity	8.83	maam	7.83	boys	9.00	males	9.00
femme	-	madam	8.33	fella	5.33	man	8.83
gal	6.83	maiden	8.67	gent	6.33	man's	8.67
gals	7.00	missus	8.67	gentleman	9.00	manhood	-
girl	8.83	ms	8.33	gentleman's	-	manly	8.67
girl's	7.00	schoolgirl	6.17	gentlemen	9.00	masculine	8.50
girlhood	7.33	schoolgirls	-	gents	7.17	masculinity	8.67
girlish	7.50	she	7.83	guy	7.33	men	9.00
girls	8.17	shes	-	guys	-	mens	8.67
girly	7.50	woman	9.00	he	9.00	mister	8.33
her	9.00	woman's	8.33	hes	8.83	mr	8.83
hers	9.00	womanhood	9.00	him	8.83	schoolboy	7.50
herself	9.00	womanly	7.50	himself	9.00	schoolboys	6.67
ladies	8.83	women	9.00	his	8.83	sir	8.33
lady	8.83	womens	-				

Note. The words that do not have ratings were added after the rating study was conducted, either because of suggestions from the coders or to parallel an other-gender counterpart already on the list as described in the Materials and Methods sections of the main text.

Study 2A

Additional Methodological Details of the Findings Reported in the Main Text in Study 2A

Our final word list consisted of 538 trait words—145 of which had gender stereotypicality designations (Table S3). These trait words have been widely used to study human personality (*48*, *82-86*). The gender words—i.e., words for WOMEN and words for MEN—were the same as in Study 1 (Table S2).

Additional Analytic Details of the Findings Reported in the Main Text in Study 2A

As reported in the main text regarding our first prediction, we found that trait words were more similar in their usage to words for MEN (M = 0.14, SD = 0.04) than to words for WOMEN (M = 0.13, SD = 0.04), B = 0.01, SE < 0.01, p < .001, d = 0.29. This result was the output of a mixed-effects linear regression with gender (words for MEN, words for WOMEN; categorical variable) predicting cosine similarity to traits, with a random intercept for each trait word.

As reported in the main text regarding our second prediction, we found that the cosine similarity of the 145 trait words (a subset of the 538 trait words) with words for MEN and, separately, with words for WOMEN depended on gender stereotypicality of the traits (i.e., there was an interaction), B = 0.02, SE < 0.01, p < .001. Specifically, there was no statistically significant difference between words for MEN and traits stereotypical of men (M = 0.14, SD = 0.04) and traits stereotypical of women (M = 0.14, SD = 0.05), B < 0.01, SE = 0.01, p = .733, d = 0.06. In contrast, words for WOMEN were more similar to traits designated as stereotypical of women (M = 0.14, SD = 0.05) than to traits stereotypical of men (M = 0.13, SD = 0.04), B = –0.02, SE = 0.01, p = .039, d = –0.34. This finding was the output of a mixed-effects linear regression with gender word (words for MEN, words for WOMEN; categorical variable), trait stereotypicality (stereotypical of men, stereotypical of women; categorical variable), and their interaction predicting cosine similarity to traits, with a random intercept for each trait word. We followed up on the significant interaction within the same model using simple slopes analyses.

Table S3

List of Trait Words in Study 2A With Gender Stereotypicality Designations

Trait	Gender	Trait	Gender		Gender	Trait	Gender
abrupt	-	eager	-	lazy	-	silent	-
absent-minded	-	earnest	-	lenient	-	simple	-
abusive	M[a]	earthy	-	lethargic	-	sincere	W
accommodating	W[b]	easygoing	M	liberal	-	skeptical	-
acquiescent	-	eccentric	-	logical	M	sloppy	-
acquisitive	-	economical	M	lonely	-	slothful	-
active	-	effervescent	-	loyal	-	sluggish	-
adaptable	-	efficient	-	lustful	W	sly	-
adventurous	M	egocentric	M	magnetic	-	smart	-
affectionate	W	egotistical	M	malleable	-	smug	M
aggressive	M	eloquent	-	manipulative	-	snobbish	-
agreeable	W	emotional	W	mannerly	-	sociable	-
aimless	-	empathic	W	masochistic	-	social	W
alert	-	energetic	-	mature	-	soft	W
aloof	-	enterprising	-	meddlesome	-	soft-hearted	-
altruistic	W	enthusiastic	-	meditative	-	solicitous	-
ambitious	M	envious	-	meek	-	somber	-
amiable	-	erratic	-	melancholy	-	sophisticated	-
analytical	-	ethical	-	mercenary	-	spirited	-
angry	-	exacting	-	merry	W	spontaneous	-
animated	-	excitable	W	meticulous	-	steady	-
antagonistic	-	exhibitionistic	-	mischievous	-	stern	-
anxious	W	explosive	M	miserly	-	stingy	-
apathetic	-	expressive	-	modest	W	straightforward	M
argumentative	-	extravagant	-	moody	W	strict	-
articulate	-	extroverted	-	moral	-	strong	M
artistic	W	exuberant	-	moralistic	-	stubborn	-
assertive	M	fair	-	morose	-	subjective	-
assured	-	fastidious	-	naive	W	submissive	W
astute	-	fault-finding	W	narrow-minded	-	suggestive	W
attractive	-	fearful	W	natural	-	superstitious	-
austere	-	fidgety	-	neat	-	surly	-
autocratic	-	finicky	-	negativistic	-	suspicious	-
autonomous	M	firm	M	negligent	-	sympathetic	W
bashful	W	flamboyant	-	nervous	-	systematic	-
belligerent	-	flexible	-	nonchalant	M	tactful	-
benevolent	-	flippant	-	noncommittal	M	tactless	-
bigoted	M	flirtatious	-	nonconforming	-	talkative	W
bitter	-	folksy	-	nonpersistent	-	temperamental	W
bland	-	foolhardy	-	nonreligious	-	tempestuous	-
blase	-	forceful	M	nosey	W	tenacious	M
boastful	M	foresighted	-	objective	-	terse	-
boisterous	-	forgetful	W	obliging	-	theatric	W
bold	M	formal	-	obsessive	-	thorough	-
bossy	-	forward	M	obstinate	-	thoughtful	-
brave	M	frank	M	open-minded	-	thoughtless	-
bright	-	fretful	-	opinionated	W	thrifty	-
brilliant	M	friendly	W	opportunistic	-	timid	W
bullheaded	M	frivolous	-	optimistic	-	tolerant	-
buoyant	-	generous	W	orderly	W	touchy	W
callous	-	genial	-	organized	W	tough	M
candid	M	glib	-	outspoken	M	traditional	M
cantankerous	-	glum	-	particular	-	tranquil	-
carefree	-	gossipy	W	passionate	-	transparent	-
careful	-	greedy	-	passionless	-	trustful	-
careless	M	gregarious	-	passive	-	truthful	-
casual	-	gruff	-	patient	-	unadventurous	-
caustic	-	grumpy	M	patronizing	M	unaffectionate	M
cautious	-	guarded	-	peaceful	-	unaggressive	-
charitable	W	gullible	W	perceptive	-	unambitious	-
cheerful	W	haphazard	-	perfectionistic	W	unassuming	-
circumspect	-	happy	-	persistent	M	unattractive	-
clever	-	happy-go-lucky	-	pessimistic	-	uncharitable	-
coarse	-	hard	-	philosophical	M	uncommunicative	-
cold	-	harsh	-	placid	-	uncompetitive	-
combative	-	hearty	-	playful	-	unconscious	-

communicative	-	helpful	W	pleasant	W	unconventional	-
compassionate	W	helpless	-	poised	W	uncooperative	-
competitive	-	high-strung	-	polite	-	uncouth	-
complex	-	homespun	-	pompous	M	uncreative	-
compliant	W	honest	-	possessive	M	uncritical	-
compulsive	-	humble	-	practical	M	undemanding	-
conceited	-	humorless	W	precise	-	undependable	-
conceitless	-	humorous	M	predictable	-	underhanded	-
conciliatory	-	hypocritical	-	prejudiced	-	understanding	-
concise	-	idealist	W	pretentious	-	unemotional	M
condescending	-	ignorant	-	prideless	-	unenergetic	-
confident	M	ill-tempered	-	principled	-	unenvious	-
conscientious	-	illogical	W	progressive	-	unexcitable	-
conservative	-	imaginative	-	prompt	-	unforgiving	-
considerate	W	imitative	-	proud	M	unfriendly	-
consistent	-	immature	M	provincial	-	ungracious	-
contemplative	-	immodest	-	prudish	W	unimaginable	-
contemptuous	-	impartial	-	punctual	-	uninhibited	-
controlling	-	impatient	-	purposeful	-	uninquisitive	-
conventional	-	imperceptive	-	quarrelsome	-	unintellectual	-
cooperative	-	impersonal	-	quiet	-	unintelligent	-
cordial	-	impertinent	-	rambunctious	M	unkind	-
cosmopolitan	-	imperturbable	-	rash	-	unmoralistic	-
courageous	M	impetuous	-	rational	M	unobservant	-
courteous	-	impolite	-	reasonable	M	unpredictable	-
cowardly	-	impractical	-	rebellious	M	unprejudiced	-
crabby	-	impudent	-	reckless	-	unpretentious	-
crafty	-	impulsive	M	refined	-	unprogressive	-
cranky	-	inarticulate	-	relaxed	-	unreflective	-
creative	-	inconsiderate	M	reliable	-	unreliable	-
critical	-	inconsistent	-	religious	-	unrestrained	-
crude	M	indecisive	W	reserved	-	unruly	-
cruel	-	indefatigable	-	respectful	-	unscrupulous	-
cultured	-	independent	M	responsible	-	unselfconscious	M
cunning	-	indirect	W	restless	-	unselfish	-
curious	-	indiscreet	-	restrained	-	unsociable	-
curt	-	individualistic	-	reverent	-	unsophisticated	-
cynical	-	indulgent	-	rigid	-	unstable	W
daring	M	industrious	-	romantic	W	unsympathetic	M
deceitful	-	inefficient	-	rough	M	unsystematic	-
decisive	-	informal	-	rude	-	untalkative	-
deep	-	informative	-	ruthless	W	unvindictive	-
defensive	-	ingenious	-	sarcastic	-	urbane	-
deliberate	-	inhibited	-	scatter-brained	-	vague	-
demanding	-	inner-directed	-	scornful	-	vain	W
demonstrative	-	innovative	M	scrupulous	-	verbal	-
dependable	-	inquisitive	-	seclusive	-	verbose	-
dependent	W	insecure	W	secretive	-	versatile	-
detached	M	insensitive	M	sedate	M	vibrant	-
devil-may-care	-	insightful	-	self-critical	-	vigilant	-
devious	M	insincere	-	self-disciplined	-	vigorous	M
dignified	-	intellectual	-	self-effacing	W	vindictive	W
diplomatic	-	intelligent	-	self-examining	-	vivacious	W
direct	M	intense	-	self-indulgent	-	volatile	W
disagreeable	-	intolerant	-	self-pity	-	warm	W
discreet	-	introspective	W	self-satisfied	-	wary	-
dishonest	-	introverted	-	self-seeking	-	wasteful	-
disorderly	M	intrusive	-	selfish	-	weak	W
disorganized	-	inventive	M	selfless	W	weariless	-
disrespectful	-	irreverent	-	sensitive	W	wise	M
distrustful	-	irritable	-	sensual	W	wishy-washy	-
docile	W	jaded	-	sentimental	W	withdrawn	-
dogmatic	-	jealous	-	serious	M	witty	-
doleful	-	jovial	-	servile	-	wordy	-
dominant	M	joyless	-	sexy	-	worldly	-
domineering	-	judicious	-	shallow	W	zealous	-
down-to-earth	-	kind	-	short-sighted	-	zestful	-
dramatic	W	knowledgeable	-	shrewd	-		
dull	-	lax	M	shy	W		

Note. Traits from reference *46*. [a] Designated as stereotypical of men. [b] Designated as stereotypical of women.

Study 2B

Additional Methodological Details of the Findings Reported in the Main Text in Study 2B

Our final word list consisted of 178 trait words with gender stereotypicality designations (Table S4). The gender words were the same as in Study 1 (Table S2).

Additional Analytic Details of the Findings Reported in the Main Text in Study 2B

As reported in the main text with respect to our first prediction, we found that, overall, trait words were more similar in their usage to words for MEN (*M* = 0.15, *SD* = 0.05) than to words for WOMEN (*M* = 0.14, *SD* = 0.05), *B* = 0.01, *SE* < 0.01, *p* < .001, *d* = 0.19. This result was the output of a mixed-effects linear regression with gender (words for MEN, words for WOMEN; categorical variable) predicting cosine similarity to traits, with a random intercept for each trait word.

As reported in the main text with respect to our second prediction, we found that the cosine similarity of the 178 trait words with words for MEN and, separately, words for WOMEN depended on gender stereotypicality of the traits (i.e., there was an interaction), *B* = 0.02, *SE* < 0.01, *p* < .001. Specifically, the was no statistically significant difference between words for MEN and traits stereotypical of men (*M* = 0.15, *SD* = 0.04) compared to traits stereotypical of women (*M* = 0.14, *SD* = 0.05), *B* < 0.01, *SE* = 0.01, *p* = .807, *d* = 0.04. In contrast, words for WOMEN were more similar to traits stereotypical of women (*M* = 0.14, *SD* = 0.05) than to traits stereotypical of men (*M* = 0.13, *SD* = 0.05), *B* = –0.01, *SE* = 0.01, *p* = .049, *d* = –0.30. This result was the output of a mixed-effects linear regression with gender word (words for MEN, words for WOMEN; categorical variable), trait stereotypicality (stereotypical of men, stereotypical of women; categorical variable), and their interaction predicting cosine similarity to traits, with a random intercept for each trait. We followed up on the significant interaction within the same model using simple slopes analyses.

Table S4

List of Trait Words in Study 2B With Gender Stereotypicality Designations

Trait	Gender	Trait	Gender	Trait	Gender
active	M[a,c]	forceful	M[g]	rigid	M[c]
adventurous	M[c]	forgiving	W[c]	robust	M[c]
affected	W[b,c]	friendly	W[g]	romantic	W[d]
affectionate	W[d]	frivolous	W[c]	self-confident	M[f]
aggressive	M[d]	fussy	W[c]	self-pitying	W[c]
ambitious	M[d]	gentle	W[f]	self-reliant	M[g]
analytical	M[e]	graceful	W[f]	self-righteous	M[g]
appreciative	W[c]	greedy	M[c]	self-sufficient	M[e]
arrogant	M[d]	gullible	W[g]	selfish	M[d]
assertive	M[d]	hardhearted	M[c]	sensitive	W[d]
athletic	M[d]	hardworking	M[d]	sentimental	W[c]
autocratic	M[c]	helpful	W[f]	serious	M[c]
bossy	M[c]	honest	W[d]	sexy	W[c]
broad-shouldered	M[f]	humorous	M[c]	sharp-witted	M[c]
capable	M[c]	imaginative	W[c]	short	W[f]
cautious	W[c]	impressionable	W[g]	show-off	M[c]
changeable	W[c]	independent	M[d]	shy	W[g]
charming	W[c]	indifferent	M[c]	small-boned	W[f]
cheerful	W[g]	individualistic	M[c]	smart	W[d]
childlike	W[g]	initiative	M[c]	soft	W[f]
clean	W[g]	innovative	M[d]	softhearted	W[c]
coarse	M[c]	intelligent	W[d]	solemn	M[g]
compassionate	W[d]	intense	M[g]	solid	M[f]
competitive	M[f]	interests wide	M[c]	sophisticated	W[c]
complaining	W[c]	inventive	M[c]	spiritual	W[g]
complicated	W[c]	jealous	M[g]	steady	M[c]
conceited	M[c]	kind	W[f]	stern	M[c]
confident	M[d]	lazy	M[c]	stingy	M[c]
confused	W[c]	leader	M[f]	stolid	M[c]
consistent	M[g]	level-headed	M[d]	strong	M[d]
controlling	M[g]	logical	M[d]	stubborn	M[d]
cooperative	W[g]	loud	M[c]	sturdy	M[f]
courageous	M[d]	loyal	W[g]	submissive	W[c]
creative	W[d]	melodramatic	W[g]	suggestive	W[c]
critical	W[d]	mild	W[c]	superstitious	W[g]
cruel	M[c]	modest	W[c]	sympathetic	W[e]
curious	W[c]	muscular	M[f]	talkative	W[c]
cynical	M[c]	naive	W[g]	tall	M[f]
dainty	W[f]	nervous	W[c]	tender	W[e]
decisive	M[d]	obnoxious	M[c]	timid	W[c]
delicate	W[f]	opinionated	M[c]	touchy	W[c]
demanding	M[d]	opportunistic	M[c]	tough	M[c]
dependable	M[g]	organized	W[d]	unambitious	W[c]
dependent	W[c]	outgoing	W[d]	understanding	W[f]
determined	M[c]	patient	W[g]	unfriendly	M[c]
disciplined	M[g]	pleasant	W[c]	unintelligent	W[c]
disorderly	M[c]	pleasure-seeking	M[c]	unscrupulous	M[c]
dominant	M[e]	polite	W[d]	unselfish	W[d]
dreamy	W[c]	possessive	M[d]	unstable	W[c]
emotional	W[d]	precise	M[c]	warm	W[f]
enterprising	M[c]	progressive	M[c]	weak	W[g]
excitable	W[g]	promiscuous	M[g]	well-built	M[f]
family-oriented	W[f]	proud	M[d]	well-dressed	W[f]
fashionable	W[f]	prudish	W[c]	well-mannered	W[d]
fault-finding	W[c]	quick	M[c]	wholesome	W[g]
fearful	W[c]	rational	M[g]	witty	M[c]
fickle	W[c]	realistic	M[c]	worrying	W[c]
flatterable	W[e]	rebellious	M[g]	yielding	W[g]
flirtatious	W[g]	reckless	M[c]		
foolish	W[c]	resourceful	M[c]		

[a] Designated as stereotypical of men. [b] Designated as stereotypical of women. Gender stereotypicality designation was taken from reference [c]*49*, [d]*40*, [e]*50*, [f]*47*, [g]*48*, but note that many traits were repeated across multiple sources.

Study 3

Additional Methodological Details of the Findings Reported in the Main Text in Study 3

The final word list consisted of 252 cases of verbs with male-biased vs. female-biased designations. Note that these 252 cases of verbs corresponded to 211 unique verbs; there were some repetitions based on differing valence or subject vs. object position of the gender bias as explained in the Materials and Methods section of the main text (Table S5). The gender words were the same as in Study 1 (Table S2).

Additional Analytic Details of the Findings Reported in the Main Text in Study 3

Regarding our first prediction, as reported in the main text, we found that verbs were overall more similar in their usage to words for MEN (M = 0.11, SD = 0.04) than to words for WOMEN (M = 0.10, SD = 0.04), B = 0.01, SE < 0.01, p < .001, d = 0.26. This result was the output of a mixed-effects linear regression with gender words (words for MEN, words for WOMEN; categorical variable) predicting cosine similarity to verbs, with a random intercept for each verb.

Regarding our second prediction, as reported in the main text, we also found that the cosine similarity of the 252 verbs with words for MEN and, separately, words for WOMEN depended on gender bias of the verbs (i.e., there was an interaction), B = 0.01, SE < 0.01, p < .001. Specifically, there was no statistically significant difference between words for MEN and verbs that were male-biased (M = 0.11, SD = 0.04) compared to verbs that were female-biased (M = 0.11, SD = 0.04), B = –0.01, SE = 0.01, p = .128, d = –0.20. In contrast, words for WOMEN were more similar to female-biased verbs (M = 0.11, SD = 0.05) than to male-biased verbs (M = 0.09, SD = 0.03), B = –0.02, SE = 0.01, p < .001, d = –0.54. This result was the output of a mixed-effects linear regression with gender word (words for MEN, words for WOMEN; categorical variable), verb syntactic bias (male-biased, female-biased; categorical variable), and their interaction predicting cosine similarity to verbs, with a random intercept for each verb. We followed up on the significant interaction within the same model using simple slopes analysis.

Table S5

List of Verbs in Study 3 with Gender-Bias Designations, Valence, and Position

Verb	Gender bias	Valence	Position of bias	Verb	Gender bias	Valence	Position of bias
adore	W[a]	positive	subject	glorify	M	positive	object
allow	M[b]	positive	subject	go	W	neutral	subject
animate	M	neutral	object	gossip	W	negative	subject
appeal	M	positive	subject	grant	M	positive	subject
appear	W	neutral	subject	greet	M	positive	object
appease	M	positive	object	harm	W	negative	subject
appoint	M	neutral	object	have	W	neutral	object
argue	M	negative	subject	have	W	neutral	subject
ask	W	neutral	object	honor	M	positive	object
assure	W	neutral	object	horrify	M	negative	subject
await	M	neutral	object	hurt	W	negative	subject
be	W	neutral	subject	incarnate	M	neutral	subject
blind	M	negative	subject	inspire	M	positive	object
bore	M	negative	object	insult	W	negative	object
brave	M	positive	object	join	M	positive	object
brave	M	positive	subject	kill	M	negative	object
bribe	M	negative	object	kill	M	negative	subject
bully	M	negative	object	kiss	W	positive	object
burn	W	neutral	object	kiss	W	positive	subject
celebrate	W	positive	subject	lament	W	negative	subject
champion	W	positive	subject	laugh	W	positive	subject
cheat	M	negative	subject	leave	W	neutral	object
clap	W	neutral	subject	like	W	positive	object
clear	M	positive	object	like	W	positive	subject
clear	M	positive	subject	live	W	positive	subject
collect	M	neutral	subject	marry	W	neutral	object
come	W	neutral	subject	marry	W	positive	subject
comfort	M	positive	subject	mature	W	positive	subject
commend	M	positive	object	meet	W	positive	object
compel	M	negative	object	meet	W	positive	subject
complain	W	negative	subject	mock	M	negative	object
concern	M	negative	subject	mourn	W	negative	subject
confess	W	negative	subject	murder	M	negative	object
congratulate	M	positive	object	murder	M	negative	subject
create	W	positive	object	neglect	M	negative	subject
create	M	neutral	subject	obscure	M	negative	subject
cry	W	negative	object	offend	M	negative	object
damn	M	negative	subject	order	M	negative	object
dance	W	positive	subject	overrun	W	negative	subject
deceive	M	negative	object	pay	M	neutral	object
defeat	M	negative	object	pay	M	neutral	subject
denounce	M	negative	object	persecute	W	negative	object
denounce	M	negative	subject	persecute	W	negative	subject
deny	M	negative	object	play	W	positive	object
depose	M	neutral	object	play	W	positive	subject
deprive	M	negative	object	pour	W	neutral	object
deprive	M	negative	subject	praise	M	positive	object
destroy	M	negative	object	praise	M	positive	subject
direct	M	neutral	object	present	W	neutral	object
dispute	M	negative	subject	present	M	neutral	subject
distract	W	negative	object	pretend	M	neutral	subject
drag	W	negative	object	prevent	M	neutral	object
dress	W	neutral	subject	promise	M	positive	subject
drown	W	negative	object	prompt	M	neutral	subject
duplicate	M	neutral	subject	prosper	M	positive	subject
elect	M	neutral	object	prostrate	M	neutral	subject
encourage	M	positive	subject	protect	W	positive	object
enrage	M	negative	object	protect	M	positive	subject
enrich	M	positive	object	protest	M	negative	subject
entertain	W	positive	object	rape	W	negative	object
equal	M	neutral	object	reach	M	neutral	object
escape	M	neutral	object	reach	M	neutral	subject
escape	M	neutral	subject	rescue	M	positive	subject
escort	W	neutral	object	respect	M	positive	object
espouse	W	neutral	object	respect	M	positive	subject

exalt	W	positive	subject	restore	M	positive	object
exalt	M	positive	object	reward	M	positive	object
excel	W	positive	object	reward	M	positive	subject
exchange	W	neutral	object	rush	M	neutral	subject
excite	M	positive	object	saw	W	neutral	object
exclaim	W	neutral	object	scare	W	negative	object
excommunicate	M	neutral	object	scold	W	negative	subject
exempt	M	neutral	object	scold	M	negative	object
expel	M	neutral	object	scream	W	negative	object
expel	M	negative	subject	scream	W	negative	subject
exploit	W	negative	object	see	W	neutral	object
expose	W	neutral	object	set	M	neutral	object
extend	W	neutral	subject	set	M	neutral	subject
extol	W	positive	subject	shame	W	neutral	object
extol	M	positive	object	shock	W	negative	object
eye	W	positive	object	shock	M	negative	subject
facilitate	W	positive	subject	shop	M	neutral	object
fade	W	neutral	object	signal	W	neutral	object
fail	M	negative	object	smile	W	positive	subject
faint	W	neutral	subject	sniff	W	neutral	subject
fall	W	neutral	subject	speak	M	neutral	object
fan	W	positive	subject	spin	W	neutral	subject
fascinate	W	positive	subject	steal	W	negative	object
fatigue	W	negative	subject	strike	M	neutral	subject
favor	M	positive	subject	strut	W	neutral	object
favour	M	positive	subject	succeed	M	positive	object
fear	M	negative	object	succeed	M	positive	subject
fear	M	negative	subject	suffer	W	negative	object
feature	W	neutral	object	summon	M	neutral	object
fee	W	neutral	subject	support	M	positive	subject
feign	W	negative	subject	surpass	W	positive	subject
felicitate	W	positive	subject	take	W	neutral	object
fell	W	neutral	subject	tarry	M	neutral	subject
fertilize	W	neutral	object	tease	W	negative	object
fertilize	W	neutral	subject	temper	M	negative	subject
fight	M	neutral	object	terrify	W	negative	object
fill	W	neutral	subject	thank	M	positive	object
find	W	neutral	subject	threaten	M	negative	subject
fit	M	positive	object	tip	M	neutral	object
fit	M	positive	subject	treat	W	positive	object
flatter	M	positive	object	treat	M	positive	subject
flourish	M	positive	subject	unmake	M	neutral	object
fly	W	neutral	subject	uphold	M	positive	object
follow	M	neutral	object	use	M	neutral	object
fondle	W	positive	object	vanish	W	neutral	subject
forbid	W	negative	object	violate	W	negative	object
forbid	M	negative	subject	visit	W	neutral	object
found	M	neutral	object	wag	M	neutral	subject
found	M	neutral	subject	want	M	neutral	subject
freeze	W	positive	subject	warm	M	positive	subject
freeze	M	neutral	subject	wear	W	neutral	subject
fright	W	negative	object	weep	W	negative	object
fright	M	negative	subject	weep	W	negative	subject
frighten	W	negative	object	welcome	M	positive	object
front	W	neutral	subject	welcome	M	positive	subject
frustrate	M	negative	subject	win	W	positive	object
gasp	W	negative	subject	win	M	positive	subject
gentle	M	positive	object	wish	W	positive	object
get	W	negative	subject	wish	M	positive	subject
giggle	W	positive	subject	woo	W	positive	object
give	W	positive	subject	worry	W	negative	subject

Note. Traits from reference *51*. [a] Designated as female-biased. [b] Designated as male-biased.

Exploratory Analyses in Study 3

The list of 252 verbs was taken from prior work that, in addition to identifying the syntactic gender bias of each verb, indicated the valence (i.e., sentiment) of the verb as positive, negative, or neutral and indicated whether the verb's gender bias occurred with arguments in the subject or object position (*51*). In two sets of exploratory analyses, we tested whether the findings in the present study were further moderated by valence or by the syntactic position in which the gender bias occurred.

Valence of the Verb. To test the potential moderating effect of valence, we first compared a mixed-effects linear regression with gender word (words for MEN, words for WOMEN; categorical variable), valence of the verb (negative, positive, or neutral; categorical variable), and their interaction terms predicting cosine similarity to verbs, with a random intercept for each verb, to an identical model that omitted the interaction terms. There was no evidence that the model with interaction terms explained significantly more variance than the model without the interaction terms, $\chi^2(2) < 0.01$, $p > .999$, indicating that valence did not moderate the difference between words for MEN and words for WOMEN.

Second, we compared a mixed-effects linear regression with gender word (words for MEN, words for WOMEN; categorical variable), verb syntactic gender bias (male-biased, female-biased; categorical variable), verb valence (negative, positive, or neutral; categorical variable), and their interaction terms predicting cosine similarity to verbs, with a random intercept for each verb, to an identical model but without the higher-order valence interaction terms. There was no evidence that the model with the valence interaction terms explained more variance, $\chi^2(6) < 0.01$, p > .999. Thus, in both of these analyses, there was no evidence that the valence of the verb moderated either the overall difference between words for MEN and words for WOMEN or the interaction effect between the gender words and the verb syntactic gender bias.

Syntactic Position of the Verb's Gender Bias. To test the potential moderating effect of the syntactic position in which the gender bias occurred for the verbs, we first conducted a mixed-effects linear regression with gender word (words for MEN, words for WOMEN; categorical variable), verb syntactic position of the bias (subject, object; categorical variable), and their interaction term predicting cosine similarity to verbs, with a random intercept for each verb. The interaction between gender and syntactic position was not significant, $B < 0.01$, $SE < 0.01$, $p = .142$, indicating that synaptic position did not moderate the difference between words for MEN and words for WOMEN.

Second, we conducted a mixed-effects linear regression with gender word (words for MEN, words for WOMEN; categorical variable), verb syntactic gender bias (male-biased, female-biased; categorical variable), syntactic position of the bias (subject, object; categorical variable), and their interaction terms. The interaction between gender word, verb syntactic gender bias, and syntactic position was not significant, $B < 0.01$, $SE = 0.01$, $p = .722$. Thus, in both of these analyses, there was no evidence that the syntactic position of the gender bias for each verb moderated either the overall difference between words for MEN and words for WOMEN or the interaction effect between the gender words and the verb syntactic gender bias.

Preregistered Replication Studies

Overview of Replication Studies

We conducted direct, preregistered replications of Studies 1-3. Each replication used identical lists of words and other procedures to Studies 1-3, respectively, with one exception: We used a different set of word embeddings. The goal of these replications was to test whether the present findings are robust to incidental details in the algorithms used to create the word embeddings.

In Studies 1-3 (main text), we used 300-dimensional fastText embeddings extracted from the Common Crawl corpus. For the present replication studies, we used 300-dimensional Global Vectors for Word Representation (GloVe) embeddings (*7*), also trained on the Common Crawl corpus.

For these replications, we preregistered our hypotheses, methods, and analytic approach, including control analyses and robustness checks reported in a subsequent section (see pp. 14-25), prior to retrieving and analyzing the word embeddings (https://osf.io/3ebqh/?view_only=feeafaf7209a4a0b9f8435273c1a4a4b).

Replication of Study 1

We compared words for PEOPLE to words for MEN and to words for WOMEN using the same mixed-effects linear regression described in Study 1. With this different set of word embeddings, we replicated Study 1 and found that words for PEOPLE were more similar in their use to words for MEN ($M = 0.19$, $SD = 0.06$) than to words for WOMEN ($M = 0.15$, $SD = 0.04$), $B = 0.04$, $SE < 0.01$, $p < .001$, $d = 0.67$.

Replication of Study 2A

To test our first prediction that, overall, trait words would be more similar in their usage to words for MEN than to words for WOMEN, we used the same multilevel model described in Study 2A. We replicated Study 2A and found that trait words were more similar in their usage to words for MEN ($M = 0.14$, $SD = 0.06$) than to words for WOMEN ($M = 0.13$, $SD = 0.06$), $B = 0.02$, $SE < 0.01$, $p < .001$, $d = 0.26$.

To test our second prediction that there would be an asymmetry in gender-stereotypical associations reflected in collective concepts, we conducted the same mixed-effects linear regression described in Study 2A. We again replicated Study 2A and found that the similarity between the words for MEN and WOMEN and the trait words depended on the gender stereotypicality of the traits (i.e., there was an interaction), $B = 0.03$, $SE < 0.01$, $p < .001$. Specifically, words for MEN did not differ significantly in their similarity to traits stereotypical of men ($M = 0.16$, $SD = 0.06$) and to traits stereotypical of women ($M = 0.16$, $SD = 0.06$), $B < 0.01$, $SE = 0.01$, $p = .650$, $d = 0.07$. In contrast, words for WOMEN were more similar to traits stereotypical of women ($M = 0.15$, $SD = 0.06$) than to traits stereotypical of men ($M = 0.13$, $SD = 0.05$), $B = –0.02$, $SE < 0.01$, $p = .033$, $d = –0.35$.

Replication of Study 2B

We note one departure from the preregistration of this replication study. The preregistration indicates that we will test 180 traits; however, in the present replication study (as in Study 2B), we analyzed 178 traits because we removed the traits “feminine” and “masculine,” which appeared in our list of gender words (Table S2). This was the only departure from the preregistration.

To test our first prediction that, overall, trait words would be more similar in their usage to words for MEN than to words for WOMEN, we used the same mixed-effects linear regression described in Study 2B. We replicated Study 2B and found that trait words were more similar to words for MEN ($M = 0.16$, $SD = 0.06$) than to words for WOMEN ($M = 0.15$, $SD = 0.06$), $B = 0.02$, $SE < 0.01$, $p < .001$, $d = 0.28$.

To test our second prediction that there would be an asymmetry in gender-stereotypical associations reflected in collective concepts, we conducted the same mixed-effects linear regression described in Study 2B. We again replicated Study 2B and found that the similarity between the words for MEN and words for WOMEN and the trait words depended on gender stereotypicality of the traits (i.e., there was an interaction), $B = 0.02$, $SE < 0.01$, $p < .001$. Specifically, words for MEN did not differ significantly in their similarity traits stereotypical of men ($M = 0.16$, $SD = 0.06$) and to traits stereotypical of women ($M = 0.17$, $SD = 0.06$), $B = –0.01$, $SE = 0.01$, $p = .237$, $d = –0.17$. In contrast, words for WOMEN were more similar to traits stereotypical of women ($M = 0.16$, $SD = 0.06$) than to traits stereotypical of men ($M = 0.13$, $SD = 0.05$), $B = –0.03$, $SE = 0.01$, $p < .001$, $d = –0.55$.

Replication of Study 3

To test our first prediction that, overall, verbs would be more similar in their usage to words for MEN than to words for WOMEN, we used the same mixed-effects linear regression described in Study 3. We replicated Study 3 and found that verbs were more similar to words for MEN (M = 0.16, SD = 0.06) than to words for WOMEN (M = 0.14, SD = 0.06), B = 0.02, SE < 0.01, p < .001, d = 0.40.

To test our second prediction that there would be an asymmetry in gender-stereotypical associations reflected in collective concepts, we conducted the same mixed-effects linear regression described in Study 3. We again replicated Study 3 and found that the similarity between the words for MEN and WOMEN and the verbs depended on the gender bias of the verbs (i.e., there was an interaction), B = 0.02, SE < 0.01, p < .001. As in Study 3, words for WOMEN were more similar to female-biased verbs (M = 0.15, SD = 0.06) than to male-biased verbs (M = 0.12, SD = 0.05), B = –0.04, SE = 0.01, p < .001, d = –0.66. Unlike Study 3, we also found that words for MEN were more similar to female-biased verbs (M = 0.17, SD = 0.06) than to male-biased verbs (M = 0.15, SD = 0.05), B = –0.02, SE = 0.01, p = .008, d = –0.35, but note that this effect for words for MEN was significantly weaker than the same effect for words for WOMEN given the significant interaction. This last finding about words for MEN is a minor departure from Study 3, but the overall pattern of results is consistent between the two studies because there was again evidence for an asymmetry in gender-stereotypical associations and specifically for stronger gender-stereotypical associations about WOMEN than about MEN in collective concepts.

Control Analyses and Robustness Checks

Overview of Control Analyses and Robustness Checks

The results of Studies 1-3 were robust to a variety of control analyses and robustness checks. These included the following, each of which is described in greater detail below and was preregistered for the replication studies: (a) in Study 1, adding weights to the analysis such that the words for PEOPLE that were rated as more representative of the concept by coders were weighted more heavily; (b) in Studies 1-3, removing *masculine generic* words and their counterparts and recomputing the analyses; (c) in Studies 1-3, conducting "leave one out" analyses for the key result; (d) in Studies 1-3, conducting a permutation test of the key result; (e) relevant to Studies 1-3, testing for potential differences in word frequencies of the gender words; and (f) in Studies 2A, 2B, and 3, conducting *word-embedding association tests* (WEAT). Finally, we also (g) tested the generalizability of the critical findings in Study 1 to a more specialized domain. We replicated the results of Study 1 in the biomedical domain using word embeddings trained on biomedical and clinical text instead of undifferentiated text on the internet (i.e., the Common Crawl, which was the basis of the studies reported in the main text).

A. Weighted Analysis (Study 1 and Replication)

We conducted a supplementary analysis in which words that were rated by coders as more fitting or representative of the concept PEOPLE were weighed more heavily in the analysis. This was done just in Study 1 because the list of words for PEOPLE was generated for the purposes of this study and was relatively small compared to the list of traits and verbs in Studies 2 and 3, respectively.

As described in detail in the Materials and Methods section of the main text, six coders who were unaware of our hypotheses rated each of the words for PEOPLE on their fit with the underlying concept. We standardized these scores, added a constant (so that they are all positive), and then used these as level-2 (i.e., PEOPLE word-level) weights in the same mixed-effects model described previously. For the two category words added after the coding step ("beings" and "group"), for which we did not have ratings of fit with the concept, we used the average rating of all PEOPLE words because weighted analyses do not permit missing weight values.

In the weighted analysis for Study 1, we again found that words for PEOPLE were more similar in their usage to words for MEN (M = 0.16, SD = 0.04) than to words for WOMEN (M = 0.14, SD = 0.04), B = 0.02, SE < 0.01, p < .001, d = 0.49. In the preregistered replication of Study 1 using this weighted analysis, we also again found that the words for PEOPLE were more similar in their usage to words for MEN (M = 0.19, SD = 0.06) than to words for WOMEN (M = 0.15, SD = 0.04), B = 0.04, SE < 0.01, p < .001, d = 0.72.

B. Masculine Generic Analyses (All Studies)

Some of the words for MEN in our list of gender words (Table S2) are also commonly used to generically refer to people of all genders. For instance, it is common when referring to a person in general to use "he" but not "she" (*27*). These words are known as masculine generics. It was important to rule out the possibility that the results we observed in the present study were merely an artifact of having these masculine generic words in our word lists, which could have artificially inflated the similarity of MEN words and PEOPLE words.

To investigate this alternative explanation, we removed masculine generic words as well as parallel woman-specific ones from our lists (i.e., "he," "hes," "him," "himself," "his," "man," and "man's"; "she," "shes," "her," "herself," "hers," "woman," and "woman's") and re-ran all analyses for Studies 1-3 and their preregistered replications. All results across all studies were robust to removing masculine generic words (for details, see Tables S6 and S7). Thus, the findings reported in the main text are not merely due to the inclusion of masculine generics among the words for MEN in our list of gender words.

Table S6

The Difference Between Gender Words in Studies 1-3 and Replications Without the Masculine Generic Words and in the Original Results

Study	Comparison	Results without masculine generic words			Original results		
		Words for MEN *M* (*SD*)	Words for WOMEN *M* (*SD*)	*d*	Words for MEN *M* (*SD*)	Words for WOMEN *M* (*SD*)	*d*
Study 1	Similarity to PEOPLE words	0.15 (0.04)	0.14 (0.03)	0.43 ***	0.16 (0.04)	0.14 (0.04)	0.47 ***
Study 1 replication		0.17 (0.05)	0.13 (0.04)	0.76 ***	0.19 (0.06)	0.15 (0.04)	0.67 ***
Study 2A	Similarity to traits	0.14 (0.05)	0.13 (0.05)	0.25 ***	0.14 (0.04)	0.13 (0.04)	0.29 ***
Study 2A replication		0.14 (0.06)	0.12 (0.05)	0.31 ***	0.14 (0.06)	0.13 (0.06)	0.26 ***
Study 2B	Similarity to traits	0.14 (0.05)	0.13 (0.05)	0.18 ***	0.15 (0.05)	0.14 (0.05)	0.19 ***
Study 2B replication		0.16 (0.06)	0.14 (0.06)	0.32 ***	0.16 (0.06)	0.15 (0.06)	0.28 ***
Study 3	Similarity to verbs	0.14 (0.05)	0.13 (0.05)	0.21 ***	0.11 (0.04)	0.10 (0.04)	0.26 ***
Study 3 replication		0.14 (0.06)	0.12 (0.06)	0.38 ***	0.16 (0.06)	0.14 (0.06)	0.40 ***

*** $p < .001$

Table S7

The Interactions Between Gender Words and Gender Stereotypicality in Studies 2 and 3 and Replications Without the Masculine Generic Words and in the Original Results

Study	Comparison	Results without masculine generic words				Original results			
		Traits stereotypical of men *M* (*SD*)	Traits stereotypical of women *M* (*SD*)	*d*	Int.	Traits stereotypical of men *M* (*SD*)	Traits stereotypical of women *M* (*SD*)	*d*	Int.
Study 2A	Similarity to words for MEN	0.14 (0.04)	0.14 (0.05)	–0.04	***	0.14 (0.04)	0.14 (0.05)	0.06	***
	Similarity to words for WOMEN	0.13 (0.04)	0.14 (0.06)	–0.37 ***		0.13 (0.05)	0.14 (0.05)	–0.34 *	
Study 2A replication	Similarity to words for MEN	0.15 (0.06)	0.16 (0.06)	–0.04	***	0.16 (0.06)	0.16 (0.06)	0.07	***
	Similarity to words for WOMEN	0.13 (0.05)	0.15 (0.07)	–0.44 ***		0.13 (0.05)	0.15 (0.06)	–0.35 ***	
Study 2B	Similarity to words for MEN	0.14 (0.05)	0.14 (0.05)	–0.03	***	0.15 (0.04)	0.14 (0.05)	0.04	***
	Similarity to words for WOMEN	0.13 (0.05)	0.14 (0.06)	–0.30 ***		0.13 (0.05)	0.14 (0.05)	–0.30 *	
Study 2B replication	Similarity to words for MEN	0.15 (0.05)	0.16 (0.06)	–0.19 ***	***	0.16 (0.06)	0.17 (0.06)	–0.17	***
	Similarity to words for WOMEN	0.12 (0.05)	0.16 (0.06)	–0.54 ***		0.13 (0.05)	0.16 (0.06)	–0.55 ***	
		Male-biased verbs *M* (*SD*)	Female-biased verbs *M* (*SD*)			Male-biased verbs *M* (*SD*)	Female-biased verbs *M* (*SD*)		
Study 3	Similarity to words for MEN	0.10 (0.04)	0.11 (0.04)	–0.24	***	0.11 (0.04)	0.11 (0.04)	–0.20	***
	Similarity to words for WOMEN	0.08 (0.03)	0.10 (0.05)	–0.51 ***		0.09 (0.03)	0.11 (0.05)	–0.54 ***	
Study 3 replication	Similarity to words for MEN	0.13 (0.05)	0.15 (0.06)	–0.39 **	***	0.15 (0.05)	0.17 (0.06)	–0.35 **	***
	Similarity to words for WOMEN	0.10 (0.05)	0.14 (0.06)	–0.66 ***		0.12 (0.05)	0.15 (0.06)	–0.66 ***	

Note. Asterisks in the “Int.” (interaction) column indicate that there was an interaction between gender words (words for MEN, words for WOMEN; categorical variable) and trait/verb gender stereotypicality (stereotypical of men, stereotypical of women; categorical variable), which provides evidence for an asymmetry in gender-stereotypical associations in collective concepts.

*** $p < .001$. ** $p < .01$. * $p < .05$

C. "Leave One Out" Analyses (All Studies)

In addition to specifically considering masculine generic words, it was important to rule out the possibility that the results of the present studies were overly reliant on any particular word. To do so, we conducted so-called "leave one out" analyses.

For these analyses, we focused on the difference in similarity between words for MEN vs. words for WOMEN and words for PEOPLE (Study 1), trait words (Studies 2A and 2B), and verbs (Study 3). (That is, we did not examine interactions with gender stereotypicality from Studies 2A, 2B, and 3.) For example, in Study 1 we re-computed the model described above 104 times, each time leaving out a single word for PEOPLE, a single word for WOMEN, or a single word for MEN. The resulting effect sizes for the difference in similarity between words for MEN vs. words for WOMEN with words for PEOPLE for each of these iterations are presented in Fig. S1. For analogous effect sizes for Studies 2A, 2B, and 3, see Figs. S2, S3, and S4, respectively. Visual inspection of these plots suggests that leaving out certain words sometimes resulted in smaller or larger effect sizes, but the effect sizes were generally quite consistent.

Fig. S1

The Difference Between Gender Words When Each Person Word and Each Gender Word is Omitted in Study 1 (Top) and its Replication (Bottom)

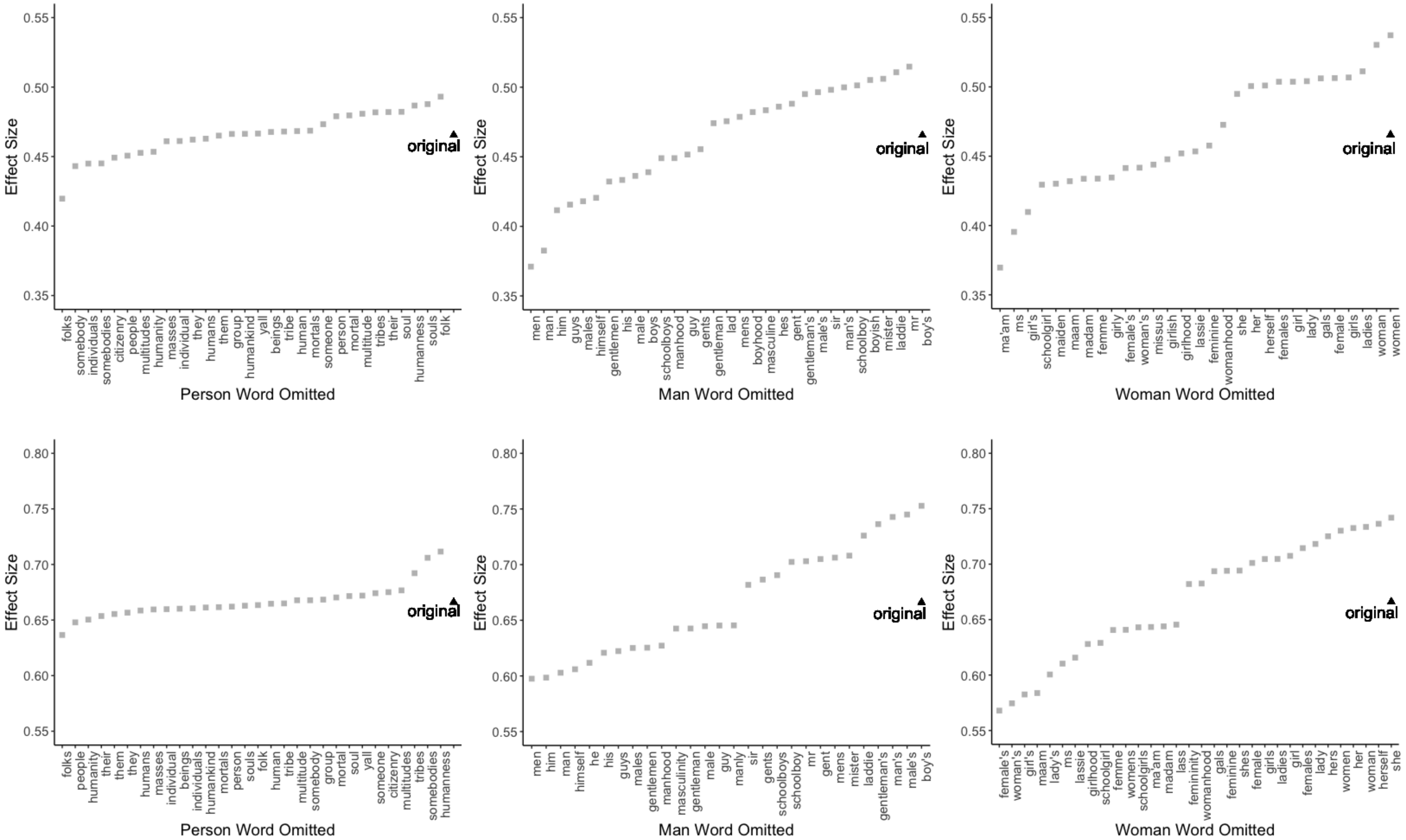

Note. “Original” refers to the magnitude of the effect size in the original model when all words were included. For readability, only gender words with the most extreme influence on the original effect size in either direction are depicted.

Fig. S2

The Difference Between Gender Words When Each Trait and Each Gender Word is Omitted in Study 2A (Top) and its Replication (Bottom)

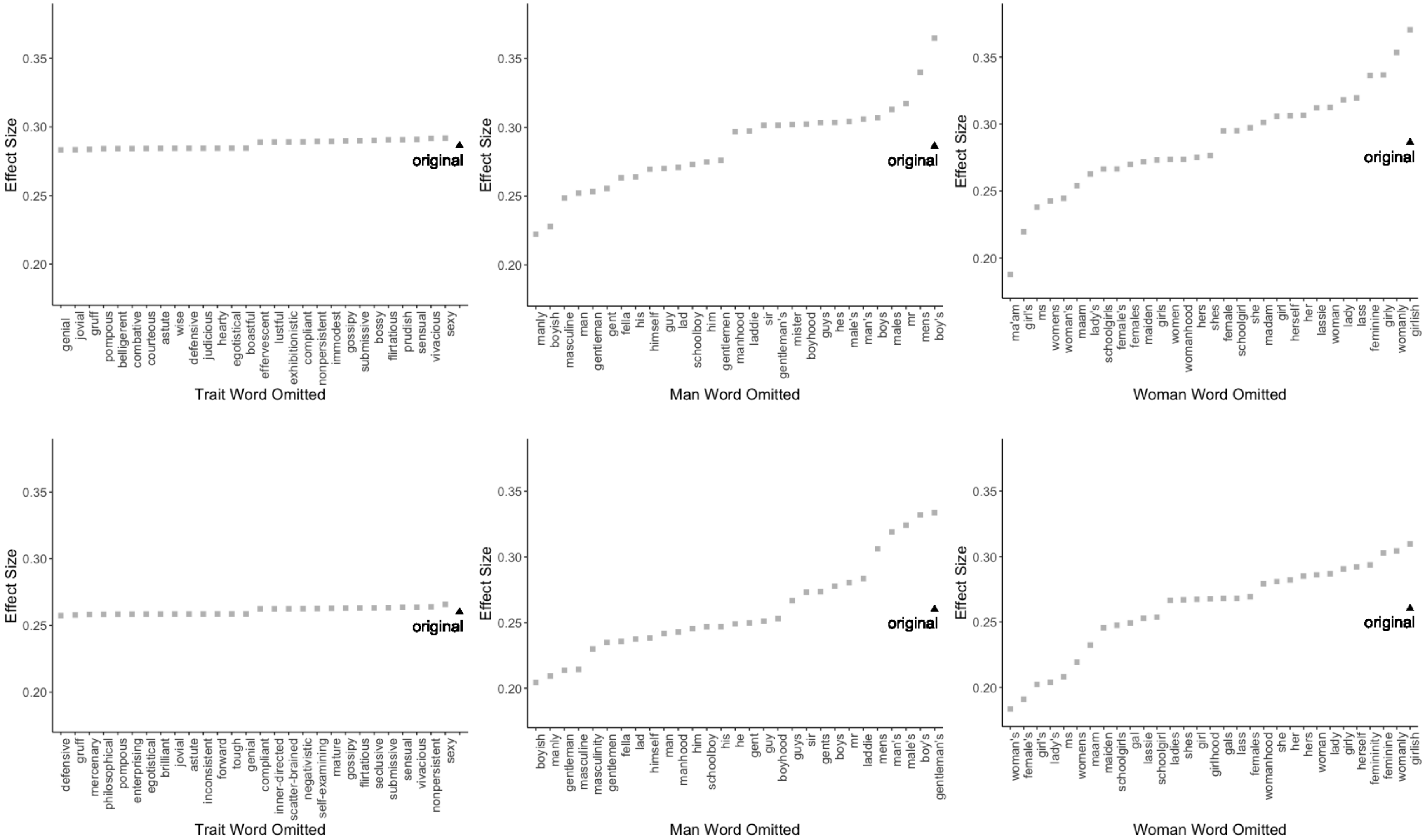

Note. "Original" refers to the magnitude of the effect size in the original model when all words were included. For readability, only gender words with the most extreme influence on the original effect size in either direction are depicted.

Fig. S3

The Difference Between Gender Words When Each Trait and Each Gender Word is Omitted in Study 2B (Top) and its Replication (Bottom)

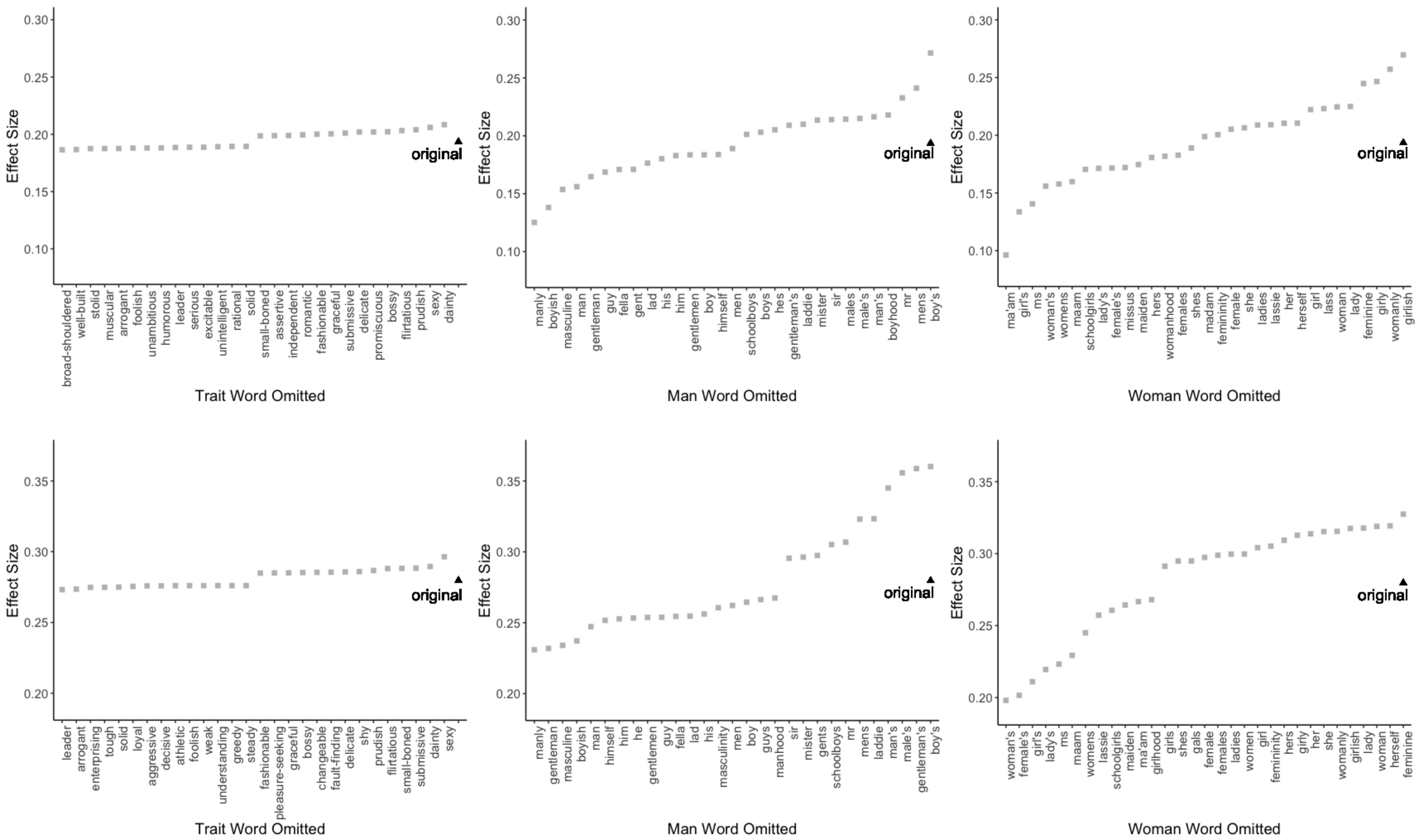

Note. "Original" refers to the magnitude of the effect size in the original model when all words were included. For readability, only gender words with the most extreme influence on the original effect size in either direction are depicted.

Fig. S4

The Difference Between Gender Words When Each Verb and Each Gender Word is Omitted in Study 3 (Top) and its Replication (Bottom)

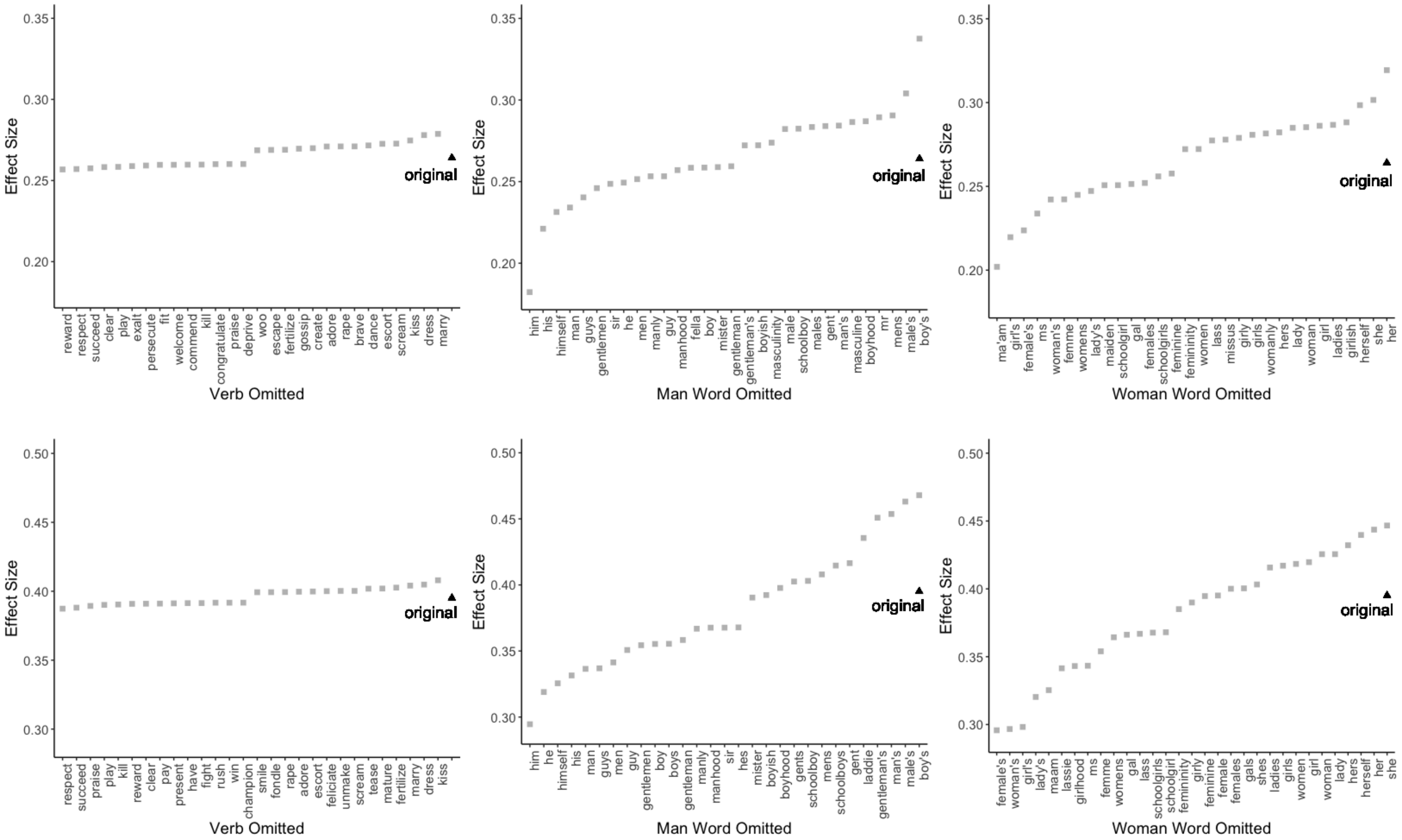

Note. “Original” refers to the magnitude of the effect size in the original model when all words were included. For readability, only gender words with the most extreme influence on the original effect size in either direction are depicted.

D. Random Permutation Tests (All Studies)

To again ensure that the results were not overly reliant on particular gender words, we also conducted random permutation tests of the key result. Permutations tests are nonparametric, and do not rely on any particular assumptions about the distribution of the data. For these analyses, we again focused on the difference in similarity between words for MEN vs. words for WOMEN and words for PEOPLE (Study 1), trait words (Studies 2A and 2B), and verbs (Study 3). (That is, we did not examine interactions with gender stereotypicality from Studies 2A, 2B, and 3.)

Taking Study 1 as an example, the permutation test involved recomputing the multilevel model described above 10,000 times, each time randomly shuffling the gender to which each word was assigned (e.g., "he" was randomly designated as a word for WOMEN or as a word for MEN). This procedure was repeated for Studies 2A, 2B, and 3.

This process created data-driven estimates of the null distributions of effect sizes and facilitated a comparison between the null distributions and the observed effects. If any particular gender word or subset of gender words was responsible for the observed effects, then the effect sizes resulting from some of the permutations would be similar to our observed effect sizes. Instead, we consistently found that our observed effect sizes were noticeably larger than the null distributions of effect sizes. Thus, these random permutation tests provide converging evidence that words for PEOPLE (Study 1), trait words (Studies 2A and 2B), and verbs (Study 3) were all more similar to words for MEN than to words for WOMEN (all *p*'s < .001 for both the main studies and replication studies).

E. Frequency Analysis of the Gender Words (All Studies)

We tested potential differences in the frequency of the words for WOMEN and the words for MEN in the training corpus (Common Crawl) used by both fastText (Studies 1-3) and GloVe (replications of Studies 1-3). Although we took care to create lists of words for WOMEN and words for MEN that were parallel in terms of their meaning and syntax, these two sets of gender words may nevertheless differ in terms of frequency. Word embeddings are somewhat sensitive to frequency (*57*), and thus it was important to consider this possibility.

To measure frequency, we used information from fastText, which provides the frequency rank of each word in the Common Crawl corpus. (GloVe does not supply frequency information, but both of these algorithms use the same corpus, so the frequency ranks should be extremely similar.) The most frequent word in the Common Crawl is ranked as 1, the next most frequent word as 2, and so on. Although this frequency information is encoded as ranks (rather than exact frequencies), this metric is relatively precise because of the massive scale of the corpus (i.e., over 630 billion word tokens). This rank data also has the benefit of being based on the same information that the word embeddings themselves were based on.

To test for potential frequency differences between our two sets of gender words, we computed a Mann-Whitney U test, which is appropriate for rank data, but found no evidence for a difference between the frequency ranks of words for MEN (*M* = 426,964.20, *SD* = 1,137,915.00, *Median* = 19,873.00) and words for WOMEN (*M* = 460,639.70, *SD* = 1,109,425.00, *Median* = 26,369.50), U = 760, *p* = .416, *d* = –0.03.

F. Word-Embedding Association Tests (Studies 2 and 3 and Replications)

Prior investigations of gender-stereotypical associations in word embeddings conducted a word-embedding association test (WEAT; *21*). This test was designed to be conceptually analogous to a common measure of human stereotypes from the psychology literature: the implicit association test (IAT; *41*). Because both the WEAT and the IAT rely on a double difference score (see details below), they obscure the asymmetry in gender-stereotypical associations we predicted and found in the present research. To compare the present data to previous investigations of gender-stereotypical associations in word embeddings, we conducted a WEAT of gender-stereotypical associations with traits and verbs in Studies 2A, 2B, and 3. We expected to conceptually replicate previous work and find evidence for gender-stereotypical associations in word embeddings.

In Studies 2A and 2B, the WEAT involves first calculating the mean similarity of each trait word to each of the words for WOMEN and, separately, each of the words for MEN and then averaging within gender set. Next, a difference score is calculated between the average similarity of each trait word with words for MEN and words for WOMEN. For traits stereotypical of women, higher difference scores would indicate *less* bias in line with gender-stereotypical associations (i.e., traits stereotypical of women are more similar to words for MEN than to words for WOMEN). For traits stereotypical of men, though, higher

difference scores would indicate *more* bias in line with gender-stereotypical associations (i.e., traits stereotypical of men are more similar to words for MEN than to words for WOMEN). The next step is to sum these difference scores for the traits stereotypical of men and, separately, for the traits stereotypical of women. The final step is then to compute a difference score of these sums. The resulting single number quantifies the extent to which the similarities between trait words and gender words are more in line with gender-stereotypical associations than not. A *p* value can then be obtained by conducting a two-tailed random permutation test.

Formally in the present case, let X represent our set of traits stereotypical of men and Y represent our set of traits stereotypical of women (called *target words* by the original authors; *23*). Let M and W represent our set of words for MEN and words for WOMEN, respectively (called *attribute words* by the original authors; *23*). Let $\cos(\vec{t}, \vec{w})$ represent the cosine of the angle between the word embedding of a given trait word and, in this case, the embedding of a given word for WOMEN. The WEAT test statistic is,

$$s(X,Y,M,W) = \sum_{x \in X} s(x,M,W) - \sum_{y \in Y} s(y,M,W)$$

where for each stereotypical trait (*t*),

$$s(t,M,W) = \text{mean}_{m \in M} \cos\left(\vec{t},\vec{m}\right) - \text{mean}_{w \in W} \cos\left(\vec{t},\vec{w}\right)$$

and the effect size (*d*) is,

$$\frac{\text{mean}_{x \in X} s\,(x,M,W) - \text{mean}_{y \in Y} \text{s}(y,M,W)}{\text{std\_dev}_{t \in X \cup Y} s(t,M,W)}$$

Applying this test to our data in Studies 2A and 2B, we found greater relative associations between words for MEN and traits stereotypical of men and words for WOMEN and traits stereotypical of women than the inverse (Table S8). We also applied this test to our data in Study 3 and to the replications of Studies 2A, 2B, and 3 and found similar results. Thus, our data are consistent with previous investigations of gender-stereotypical associations in word embeddings.

For instance, reference *21* found that women are associated with the arts and men are associated with the sciences compared to the inverse set of associations (*d* = 1.24). Similarly, we found that women were associated with certain female-stereotypical traits and verbs (e.g., “compassionate”) and men were associated with certain male-stereotypical traits and verbs (e.g., “brave”) more than the inverse (*d* range: 0.64-0.89). Crucially, our analyses in the main text show that *gender-stereotypical associations were driven by associations about women, not men*. Because the WEAT relies on two difference scores, it obscures the asymmetry that we predicted and found.

Table S8

WEAT Statistics in Studies 2 and 3 and Replications

Study	Target words	Attribute words	WEAT	*d*
Study 2A	Traits stereotypical of men vs. traits stereotypical of women	Words for MEN vs. words for WOMEN	1.30***	0.67
Study 2A Replication			1.81***	0.89
Study 2B			1.41***	0.57
Study 2B Replication			2.03***	0.75
Study 3	Male-biased verbs vs. female-biased verbs		1.68***	0.64
Study 3 Replication			2.14***	0.73

****p* < .001

G. Replication of Study 1 in the Biomedical Domain

We conducted another replication of Study 1 using identical lists of words and other procedures to Study 1, with one exception: We used a different set of word embeddings (*53*). The goal of this replication was to test the generalizability of the critical PEOPLE = MEN finding from Study 1 in the biomedical domain.

Similar to the word embeddings analyzed in the main text, these biomedical word embeddings were extracted with the fastText algorithm with 200 dimensions (*6*). But rather than being trained on an undifferentiated corpus of 630+ billion words on the internet (i.e., the Common Crawl corpus), the biomedical embeddings were trained on a smaller corpus of biomedical text: specifically, 4+ billion words from abstracts and titles in the PubMed biomedical and life science research archive and 500+ million words in the MIMIC-III Clinical Database of de-identified hospital clinical notes (vital sign measurements, laboratory test results, procedures, medications, etc.; *87*). The biomedical domain was of particular interest because biomedical research and clinical practice have direct implications for gender (in)equity in health, and it would thus be particularly troubling to find a PEOPLE = MEN bias in this domain.

We compared words for PEOPLE to words for MEN and to words for WOMEN using the same mixed-effects linear regression described in Study 1. Replicating Study 1, we found that words for PEOPLE were more similar in their use to words for MEN (M = 0.08, SD = 0.06) than to words for WOMEN (M = 0.05, SD = 0.04), B = 0.03, SE < 0.01, p < .001, d = 0.49. The effect size in this biomedical domain (i.e., d = 0.49) is similar to that in Study 1 reported in the main text (i.e., d = 0.47), demonstrating the generalizability of the present finding to this different domain based on a different corpus.

REFERENCES AND NOTES

1. Z. S. Harris, Distributional structure. *Word* **10**, 146–162 (1954).

2. A. Lenci, Distributional models of word meaning. *Annu. Rev. Linguist.* **5**, 151–171 (2018).

3. Y. Bengio, R. Ducharme, P. Vincent, C. Janvin, A neural probabilistic language model. *J. Mach. Learn. Res.* **3**, 1137–1155 (2003).

4. R. Collobert, J. Weston, A unified architecture for natural language processing: Deep neural networks with multitask learning, in *Proceedings of the 25th International Conference on Machine Learning* (2008), pp. 160–167.

5. T. K. Landauer, S. T. Dumais, A solution to Plato's problem: The latent semantic analysis theory of acquisition, induction, and representation of knowledge. *Psychol. Rev.* **104**, 211–240 (1997).

6. T. Mikolov, I. Sutskever, K. Chen, G. S. Corrado, J. Dean, Distributed representations of words and phrases and their compositionality, in *Advances in Neural Information Processing Systems* (2013), pp. 3111–3119.

7. J. Pennington, R. Socher, C. D. Manning, Glove: Global vectors for word representation, in *Proceedings of the 2014 Conference on Empirical Methods in Natural Language Processing* (2014), pp. 1532–1543.

8. E. Durkheim, *The Elementary Forms of Religious Life* (Oxford Univ. Press, 1915)

9. S. Moscovici, Attitudes and opinions. *Annu. Rev. Psychol.* **14**, 231–260 (1963).

10. B. K. Payne, H. A. Vuletich, K. B. Lundberg, The bias of crowds: How implicit bias bridges personal and systemic prejudice, *Psychol. Inq.* **4**, 233–248 (2017).

11. T. E. Charlesworth, V. Yang, T. C. Mann, B. Kurdi, M. R. Banaji, Gender stereotypes in natural language: Word embeddings show robust consistency across child and adult language corpora of more than 65 million words. *Psychol. Sci.* **32**, 218–240 (2021).

12. H. Ritchie, M. Roser, “Gender ratio” (Our World in Data, 2019); https://ourworldindata.org/gender-ratio.

13. T. Mikolov, E. Grave, P. Bojanowski, C. Puhrsch, A. Joulin, Advances in pre-raining distributed word representations, in *Proceedings of the Eleventh International Conference on Language Resources and Evaluation* (2018).

14. B. M. Lake, G. L. Murphy, Word meaning in minds and machines. *Psychol. Rev.* (2021).

15. J. R. Firth, A synopsis of linguistic theory, 1930–1955, in *Studies in Linguistic Analysis* (Blackwell, 1957).

16. S. McDonald, M. Ramscare, Testing the distributional hypothesis: The influence of context on judgments of semantic similarity, in *Proceedings of the Annual Meeting of the Cognitive Science Society* (2001).

17. L. Gutiérrez, B. Keith, A systematic literature review on word embeddings, in *International Conference on Software Process Improvement* (2019), pp. 132–141.

18. A. Rogers, O. Kovaleva, A. Rumshisky, A primer in BERTology: What we know about how BERT works. *Trans. Assoc. Comput.* **8**, 842–866 (2020).

19. S. Ruder, I. Vulić, A. Søgaard, A survey of cross-lingual word embedding models. *J. Artif. Intell. Res.* **65**, 569–631 (2019).

20. D. L. Rohde, L. M. Gonnerman, D. C. Plaut, An improved model of semantic similarity based on lexical co-occurrence. *Commun. ACM* **8**, 627–633 (2006).

21. A. Caliskan, J. J. Bryson, A. Narayanan, Semantics derived automatically from language corpora contain human-like biases. *Science* **356**, 183–186 (2017).

22. N. Garg, L. Schiebinger, D. Jurafsky, J. Zou, Word embeddings quantify 100 years of gender and ethnic stereotypes. *Proc. Natl. Acad. Sci. U.S.A.* **115**, E3635–E3644 (2018).

23. M. Lewis, G. Lupyan, Gender stereotypes are reflected in the distributional structure of 25 languages. *Nat. Hum. Behav.* **4**, 1021–1028 (2020).

24. S. L. Bem, *The Lenses of Gender: Transforming the Debate on Sexual Inequality* (Yale Univ. Press, 1993).

25. S. de Beauvoir, *The Second Sex*, C. Borde, S. Malovany-Chevallier, Transl. (Vintage Books, 1949/2010).

26. C. P. Gilman, *The Man-Made World: Or, Our Androcentric Culture* (Charlotte Company, ed. 3, 1911).

27. M. Hellinger, H. Bußmann, *Gender Across Languages: The Linguistic Representation of Women and Men* (John Benjamins, 2003), vol. 3.

28. A. H. Eagly, M. E. Kite, Are stereotypes of nationalities applied to both women and men? *J. Pers. Soc. Psychol.* **53**, 451–462 (1987).

29. M. C. Hamilton, Masculine bias in the attribution of personhood: People = male, male = people. *Psychol. Women Q.* **15**, 393–402 (1991).

30. R. D. Merritt, C. J. Kok, Attribution of gender to a gender-unspecified individual: An evaluation of the people = male hypothesis. *Sex Roles* **33**, 145–157 (1995).

31. A. H. Bailey, M. Lafrance, Who counts as human? Antecedents to androcentric behavior. *Sex Roles* **76**, 682–693 (2016).

32. A. H. Bailey, M. LaFrance, J. F. Dovidio, Implicit androcentrism: Men are human, women are gendered. *J. Exp. Soc. Psychol.* **89**, 103980 (2020).

33. A. H. Bailey, M. LaFrance, J. F. Dovidio, Is man the measure of all things? A social cognitive account of androcentrism. *Pers. Soc. Psychol. Rev.* **23**, 307–331 (2019).

34. M. Gustafsson Sendén, T. Lindholm, S. Sikström, Biases in news media as reflected by personal pronouns in evaluative contexts. *Soc. Psychol.* **45**, 103–111 (2014).

35. J. M. Twenge, W. K. Campbell, B. Gentile, Male and female pronoun use in U.S. books reflects women's status, 1900–2008. *Sex Roles* **67**, 488–493 (2012).

36. C. Criado-Perez, *Invisible Women: Exposing Data Bias in a World Designed for Men* (Abrams Press, 2019).

37. M. Dusenbery, *Doing Harm: The Truth About How Bad Medicine and Lazy Science Leave Women Dismissed, Misdiagnosed, and Sick* (HarperCollins, 2018).

38. P. Hegarty, O. Parslow, Y. G. Ansara, F. Quick, Androcentrism: Changing the landscape without leveling the playing field, in *The Sage Handbook of Gender and Psychology*, M. K. Ryan, N. R. Branscombe, Eds. (Sage, 2013), pp. 29–44.

39. B. A. Nosek, F. L. Smyth, J. J. Hansen, T. Devos, N. M. Lindner, K. A. Ranganath, C. T. Smith, K. R. Olson, D. Chugh, A. G. Greenwald, M. R. Banaji, Pervasiveness and correlates of implicit attitudes and stereotypes. *Eur. Rev. Soc. Psychol.* **18**, 36–88 (2007).

40. A. H. Eagly, C. Nater, D. I. Miller, M. Kaufmann, S. Sczesny, Gender stereotypes have changed: A cross-temporal meta-analysis of U.S. public opinion polls from 1946 to 2018. *Am. Psychol.* **75**, 301–315 (2020).

41. "May 2017 Crawl Archive Now Available" (Common Crawl, 2017); http://commoncrawl.org/2017/06/.

42. M. A. Mehmood, H. M. Shafiq, A. Waheed, Understanding regional context of World Wide Web using common crawl corpus, in *Proceedings of the IEEE 13th Malaysia International Conference on Communications (MICC)* (IEEE, 2017), pp. 164–169.

43. J. Dodge, M. Sap, A. Marasovic, W. Agnew, G. Ilharco, D. Groeneveld, M. Mitchell, M. Gardner, Documenting large webtext corpora: A case study on the colossal clean crawled corpus. arXiv:2104.08758 [cs.CL] (18 April 2021).

44. J. Cohen, A power primer. *Psychol. Bull.* **112**, 155–159 (1992).

45. A. Lovakov, E. R. Agadullina, Empirically derived guidelines for effect size interpretation in social psychology. *Eur. J. Soc. Psychol.* **51**, 485–504 (2021).

46. G. Saucier, K. Iurino, High-dimensionality personality structure in the natural language: Further analyses of classic sets of English-language trait-adjectives. *J. Pers. Soc. Psychol.* **119**, 1188–1219 (2020).

47. E. L. Haines, K. Deaux, N. Lofaro, The times they are a-changing… or are they not? A comparison of gender stereotypes, 1983–2014. *Psychol. Women Q.* **40**, 353–363 (2016).

48. D. A. Prentice, E. Carranza, What women and men should be, shouldn't be, are allowed to be, and don't have to be: The contents of prescriptive gender stereotypes. *Psychol. Women Q.* **26**, 269–281 (2002).

49. J. E. Williams, D. L. Best, *Measuring Sex Stereotypes: A Multination Study* (Sage, 1990).

50. S. L. Bem, The measurement of psychological androgyny. *J. Consult. Clin. Psychol.* **42**, 155–162 (1974).

51. A. Hoyle, W. Sonkin, H. Wallach, I. Augenstein, R. Cotterell, Unsupervised discovery of gendered language through latent-variable modeling. arXiv:1906.04760 [cs.CL] (11 June 2019).

52. Y. Zhang, Q. Chen, Z. Yang, H. Lin, Z. Lu. BioWordVec, improving biomedical word embeddings with subword information and MeSH. *Sci. Data* **6**, 52 (2019).

53. D. DeFranza, H. Mishra, A. Mishra, How language shapes prejudice against women: An examination across 45 world languages. *J. Pers. Soc. Psychol.* **119**, 7–22 (2020).

54. A. G. Greenwald, D. E. McGhee, J. L. Schwartz, Measuring individual differences in implicit cognition: The implicit association test. *J. Pers. Soc. Psychol.* **74**, 1464–1480 (1998).

55. "Facts and figures: Leadership and political participation" (UN Women, 2017); www.unwomen.org/en/what-we-do/leadership-and-political-participation/facts-and-figures.

56. J. Mu, S. Bhat, P. Viswanath, All-but-the-top: Simple and effective postprocessing for word representations, in *Proceedings of the 6th International Conference on Learning Representations* (2018).

57. R. T. Anderson, *Transgender Ideology Is Riddled with Contradictions* (The Heritage Foundation, 2018); www.heritage.org/gender/commentary/transgender-ideology-riddled-contradictions-here-are-the-big-ones.

58. A. Byrne, Are women adult human females? *Philos. Stud.* **177**, 3783–3803 (2020).

59. R. Dembroff, Beyond binary: Genderqueer as critical gender kind. *Philos. Impr.* **20**, 1–23 (2020).

60. K. Crenshaw, Mapping the margins: Intersectionality, identity politics, and violence against women of color. *Stanford Law Rev.* **43**, 1241–1299 (1991).

61. V. Purdie-Vaughns, R. P. Eibach, Intersectional invisibility: The distinctive advantages and disadvantages of multiple subordinate-group identities. *Sex Roles* **59**, 377–391 (2008).

62. S. G. S. Shah, R. Dam, M. J. Milano, L. D. Edmunds, L. R. Henderson, C. R. Hartley, O. Coxall, P. V. Ovseiko, A. M. Buchan, V. Kiparoglou, Gender parity in scientific authorship in a National Institute for Health Research Biomedical Research Centre: A bibliometric analysis. *BMJ Open* **11**, e037935 (2021).

63. E. H. Wojcik, J. R. Saffran, Toddlers encode similarities among novel words from meaningful sentences. *Cognition* **138**, 10–20 (2015).

64. J. R. Saffran, R. N. Aslin, E. L. Newport, Statistical learning by 8-month-old infants. *Science* **274**, 1926–1928 (1996).

65. P. Nayak, Understanding searches better than ever (Google, 2019); https://blog.google/products/search/search-language-understanding-bert/.

66. "Embeddings@Twitter" (Twitter, Revenue Platform, 2018); https://blog.twitter.com/engineering/en_us/topics/insights/2018/embeddingsattwitter.

67. A. Renduchintala, A. Williams, Investigating failures of automatic translation in the case of unambiguous gender. arXiv:2104.07838 [cs.CL] (16 April 2021).

68. E. Dinan, A. Fan, A. Williams, J. Urbanek, D. Kiela, J. Weston, Queens are powerful too: Mitigating gender bias in dialogue generation, in *Proceedings of the 2020 Conference on Empirical Methods in Natural Language Processing (EMNLP)* (2020), pp. 8173–8188.

69. C. Metz, AI is transforming Google search. The rest of the web is next (WIRED Magazine, 2016); www.wired.com/2016/02/ai-is-changing-the-technology-behind-google-searches/.

70. P. Olson, The algorithm that helped Google Translate become sexist (Forbes, 2018); www.forbes.com/sites/parmyolson/2018/02/15/the-algorithm-that-helped-google-translate-become-sexist/?sh=4de9491b7daa.

71. G. Stanovsky, N. A. Smith, L. Zettlemoyer, Evaluating gender bias in machine translation, in *Proceedings of the 57th Annual Meeting of the Association for Computational Linguistics* (2019), pp. 1679–1684.

72. A. Renduchintala, D. Diaz, K. Heafield, X. Li, M. Diab, Gender bias amplification during speed-quality optimization in neural machine translation, in *Proceedings of the 59th Annual Meeting of the Association for Computational Linguistics and the 11th International Joing Conference on Natural Language Processing* (Volumne 2: Short Paper) (2021).

73. H. Gonen, Y. Goldberg, Lipstick on a pig: Debiasing methods cover up systematic gender biases in word embeddings but do not remove them, in *Proceedings of the 2019 Conference of the North American Chapter of the Association for Computational Linguistics: Human Language Technologies*, Volume 1 (Long and Short Papers) (2019).

74. R. H. Maudslay, H. Gonen, R. Cotterell, S. Teufel, It's all in the name: Mitigating gender bias with name-based counterfactual data substitution, in *Proceedings of the 2019 Conference on*

Empirical Methods in Natural Language Processing and the 9th International Joint Conference on Natural Language Processing (2019).

75. J. W. Pennebaker, R. J. Booth, R. L. Boyd, M. E. Francis, *Linguistic Inquiry and Word Count: LIWC2015* (Pennebaker Conglomerates, 2015); www.LIWC.net.

76. F. Glen, K. Hurrell, *Technical Note: Measuring Gender Identity* (Equality and Human Rights Commission, 2012).

77. S. M. van Anders, N. L. Caverly, M. M. Johns, Newborn bio/logics and US legal requirements for changing gender/sex designations on state identity documents. *Fem. Psychol.* **24**, 172–192 (2014).

78. C. Felbaum, "About WordNet" [WordNet, Princeton University].

79. T. K. Koo, M. Y. Li, A guideline of selecting and reporting intraclass correlation coefficients for reliability research. *J. Chiropr. Med.* **15**, 155–163 (2016).

80. L. R. Goldberg, From Ace to Zombie: Some explorations in the language of personality. *J. Pers. Assess.* **1**, 203–234 (1982).

81. A. Kuznetsova, P. B. Brockhoff, R. H. B. Christensen, lmerTestPackage: Tests in linear mixed effects models. *J. Stat. Softw.* **82**, 1–26 (2017).

82. B. de Raad, D. P. Barelds, E. Levert, F. Ostendorf, B. Mlačić, L. D. Blas, M. Herbícková, Z. Szirmák, M. Perugini, A. T. Church, M. S. Katigbak. Only three factors of personality description are fully replicable across languages: A comparison of 14 trait taxonomies. *J. Pers. Soc. Psychol.* **98**, 160–173 (2010).

83. L. R. Goldberg, An alternative "description of personality": The big-five factor structure. *J. Pers. Soc. Psychol.* **59**, 1216–1229 (1990).

84. L. R. Goldberg, The development of markers for the Big-Five factor structure. *Psychol. Assess.* **4**, 26–42 (1992).

85. W. K. Hofstee, B. De Raad, L. R. Goldberg, Integration of the big five and circumplex approaches to trait structure. *J. Pers. Soc. Psychol.* **63**, 146–163 (1992).

86. G. Saucier, L. R. Goldberg, The language of personality: Lexical perspectives on the five-factor model, in *The Five-Factor Model of Personality: Theoretical Perspectives*, J. S Wiggins, Ed. (Guilford Press, 1996), pp. 21–50.

87. A. E. W. Johnson, T. J. Pollard, L. Shen, L. H. Lehman, M. Feng, M. Ghassemi, B. Moody, P. Szolovits, L. A. Celi, R. G. Mark, MIMIC-III, a freely accessible critical care database. *Sci. Data* **3**, 160035 (2016).
